# Computational Simulation of Irradiation-Induced Structural Defects in Metallic Materials: Formation, Evolution, and Mechanical Effects

**DOI:** 10.3390/nano16150914

**Published:** 2026-07-24

**Authors:** Xiang Hou, Liang Zhang, Xiaoxu Huang

**Affiliations:** 1 International Joint Laboratory for Light Alloys (MOE), College of Materials Science and Engineering, Chongqing University, Chongqing 400044, China; 2 State Key Laboratory of Mechanical Transmission for Advanced Equipment, Chongqing University, Chongqing 400044, China

**Keywords:** defect evolution, radiation damage, computational simulation, metallic materials, mechanical properties

## Abstract

The rapid development of Generation IV nuclear reactors has imposed stringent requirements on structural materials, demanding excellent irradiation resistance to withstand long-term exposure to complex radiation environments, including neutron and ion irradiation. Under irradiation, a large number of defects are generated inside materials via displacement cascades, and the dynamic evolution of these defects gradually leads to macroscopic property deterioration, potentially triggering major accidents such as equipment failure and even posing system safety hazards. Thus, understanding the law of defect evolution in materials under irradiation and exploring the microscopic mechanism of irradiation damage are core prerequisites for material service life prediction, radiation resistance optimization, and safety risk assessment. In recent years, computational simulation, leveraging its unique advantages in multiscale and multiphysics coupling research, has yielded numerous innovative achievements in the irradiation field. This review overviews the progress of computational simulation studies on irradiation damage in nuclear structural materials over the past few decades, focuses on summarizing the “generation-evolution-annihilation” process of irradiation defects, and further discusses the impact of irradiation on the macroscopic mechanical properties of materials. The content and outlook of this review can advance the microscopic-level comprehension of irradiation damage mechanisms in structural materials and provide guidance for the development of a new generation of materials with excellent irradiation resistance.

## 1. Introduction

In the nuclear power industry, high-energy particles generated in fusion reactors collide with the lattice atoms of materials, causing structural changes and the accumulation of defects at the atomic scale, resulting in degradation in the mechanical properties of materials, known as radiation damage. Primary displacement cascade is the initial stage of radiation damage, which involves the collision between high-energy particles (such as electrons [1], ions [2], dual-ion [3] and neutrons [4], or more exotic particles such as neutrinos [5]) and material atoms and produces a large number of defects, which is the key to understand the mechanism of radiation damage [6,7]. Ni-based alloys, Fe-Cr-Al alloys, W, single-phase solid solution alloys, which include high-entropy alloys, etc., all show remarkable mechanical, corrosion resistance, and high-temperature resistance, and are considered highly promising materials in fourth-generation nuclear power plants [8,9,10,11], while their radiation resistance characteristics still need further research. In metallic materials, energetic particle irradiation triggers displacement cascades that generate primary point defects (e.g., vacancies and self-interstitials). Through subsequent migration and interaction with microstructural sinks, these elementary building blocks agglomerate into complex extended structures, such as dislocation loops, voids, and precipitates. Ultimately, the long-term evolution of this defect network dictates macroscopic mechanical degradation, including swelling, hardening, and embrittlement. Despite a series of prior experimental studies on the irradiation of nuclear structural materials [12,13,14], limitations persist in terms of timescale and spatial resolution, making it difficult to precisely understand the irradiation damage mechanism at the atomic level, which restricts the development of new-generation radiation-resistant materials. Over the past few decades, atomic simulation has gradually developed, providing more abundant information for exploring the process and mechanism of irradiation damage at the atomic scale.

In the early 1950s, research on radiation damage was mainly based on simple theoretical models, such as the collision theory of classical physics [15]. Researchers simulated the interaction between irradiated particles and material atoms by assuming that the material atoms are rigid spheres and using the principle of elastic collision [16]. In 1955, the Kinchin–Pease model was proposed, which quantitatively calculated the displacement threshold energy for the first time [17]. During this period, people initially realized that collisions between irradiated particles and material atoms could cause atomic displacement and defect formation, which could reduce the electrical conductivity of the material [18]. Sigmund proposed the displacement damage theory in 1962, which laid the physical foundation for radiation damage analysis. The relationship between irradiation defect concentration *N_d_* and energy deposition *E* is as follows:

(1) $$N_{d}=\frac{\sigma }{\varepsilon }\int_{0}^{E_{max}}\frac{dN\left(E\right)}{dE}\cdot \frac{1}{1+\alpha E}dE$$

The quantitative prediction of defect concentration was achieved for the first time through the integration of elastic collision cross-sections, where σ is the atomic displacement cross-section, α is the dimensionless correction factor (probability of defect cluster formation), and $\frac{dN\left(E\right)}{dE}$ is the incident particle energy spectrum. At this stage, the theoretical model not only ignores the electron cloud interactions and lattice vibrations between atoms but also overlooks complex processes such as defect accumulation and interactions caused by multiple collisions.

In the 1970s, the molecular dynamics (MD) method began to be applied to the simulation of material irradiation damage. Norgett et al. [19] proposed the Norgett–Robinson–Torrens model in 1975 (introduced in Section 2.1), which provides macroscopic dose parameters to enable MD simulations to more clearly reveal the defect evolution mechanism during irradiation processes. Due to the limited accuracy of the potential function of the interaction at this time, complex chemical bonds and electronic effects cannot be accurately described. The improvement of potential function accuracy and multiscale expansion were the two core breakthrough directions in simulation computing. In 1984, Daw et al. [20] proposed the Embedded Atomic Method (EAM) potential, which considers the influence of electron density on interatomic interactions and has become the cornerstone of metal irradiation damage simulation. In the same year, the Finnis–Sinclair potential was developed and widely used for simulating dislocation motion and radiation-induced defect aggregation [21]. Smith et al. [22] combined EAM potential with Ziegler–Biersack–Littmark (ZBL) potential to accurately investigate the mechanism of irradiation-induced swelling in uranium alloys. In 1989, Baskes et al. [23] introduced an angle-dependent term based on EAM potential, forming a modified embedded atom method (MEAM) that is suitable for complex crystal structures such as semiconductors and oxides. Limited by the computational scale, only a thousand-atom system can be simulated at present.

With the advent of the Large-scale Atomic/Molecular Massively Parallel Simulator (LAMMPS), researchers made numerous significant advances in atomic-scale simulations of irradiation damage. In 1995, Plimpton et al. [24] simulated the defect formation process of metal bombarded by high-energy particles through LAMMPS and verified its feasibility in radiation damage simulation. Subsequent to this, LAMMPS was widely used in the simulation of ferroalloys [25], nickel alloys [26], and nanocrystalline materials [27], which accelerated the research progress of radiation damage. However, the limitations of MD simulations in describing highly non-equilibrium defect structures are due to their reliance on empirical potentials. Malerba et al. [28] critically pointed out the influence of different empirical potentials on the outcomes of irradiation simulations and advocated for the necessity of using potentials calibrated against density functional theory (DFT) data. The proposal of the Car–Parrinello method [29] and the introduction of electron-density gradient corrections [30] spurred the development of DFT in radiation damage studies to calibrate these empirical potentials and ensure accurate large-scale simulations.

Entering the 21st century, a surge of atomistic simulation methods emerged for radiation damage, later evolving into multiscale-coupled computational strategies. The core advantage of multiscale methods lies in the balance between cross-scale accuracy and efficiency. In 2004, Domain et al. [25] applied the Object Kinetic Monte Carlo (KMC) method, built upon the foundation of kinetic Monte Carlo KMC, to specific nuclear systems to enable coupling with MD for investigating radiation damage in materials. However, the spatial scale and irradiation dose (typically much less than 1 dpa) that the KMC can simulate are often constrained. In 2005, Uberuaga et al. [31] applied the temperature-accelerated dynamics framework (TAD) to radiation damage simulation, demonstrating its advantages in tracking long-term defect mobility and overcoming timescale limitations of conventional methods. Building upon the classic Mean Field Rate Theory framework originating in the 1970s, Stoller et al. [32] utilized modern parameterizations in 2008 to investigate microstructural evolution under high doses reaching 100 dpa or higher. To more accurately simulate the motion of dislocations, three-dimensional discrete dislocation dynamics (3D-DDD) has been developed, which can simulate dislocation movement under the influence of irradiation defects [33], such as dislocation loops [34,35] or stacking fault tetrahedra [36,37]. In 2018, Liu et al. [38] applied the parallel replica accelerated molecular dynamics (AMD) framework to radiation damage simulation, demonstrating its advantages in expanding simulation timescales beyond MD and capturing long-term intergranular helium bubble growth. In 2019, Wang et al. [39] applied the Deep Potential MD framework to radiation damage simulation, demonstrating its advantages in handling complex interatomic interactions and long-timescale simulations. With the centralization and intelligence of computer processing, machine learning potentials (MLPs) have been developed to fit DFT or MD data via machine learning algorithms for constructing high-precision and efficient interatomic potentials. In 2023, Liu et al. [40] utilized machine learning potentials to integrate the neuroevolution potential (NEP) framework with Ziegler–Biersack–Littmark (ZBL) screened nuclear repulsion potential, trained a NEP-ZBL framework for W, and demonstrated its accuracy in simulating defect energetics. Bhardwaj et al. [41] compared the results of radiation damage simulations in W using MLP and EAM potentials. They found that the dislocation types and densities obtained from MLP simulations are in excellent agreement with those observed experimentally, validating the advantages of MLPs in atomic-scale simulations. Recently, combining MLP-MD with DFT, Li et al. [42] developed a multiscale framework to systematically investigate the effects of grain boundaries on irradiation damage and microcrack propagation.

Several seminal reviews have laid the groundwork for this field, offering complementary and relatively specialized scopes. Nordlund et al. [43] focused primarily on the physical understanding and analytical models of primary radiation damage, whereas Nordlund [44] traced the historical development of computational methods for radiation effects. Zhang [45] emphasized grain-boundary-mediated defect annihilation and self-healing in nanocrystalline metals, while Frydrych [46] reviewed the incorporation of irradiation effects into crystal plasticity formulations. Recent milestone reviews by Ali et al. [47] summarized primary radiation damage models and SRIM-based damage predictions, whereas the corresponding complete dynamic evolution pathway of defects from an atomistic simulation viewpoint warrants elucidation. Furthermore, Fan [48] evaluated the evolution of irradiation-induced defects in terms of atomistic simulation methods, potential energy surfaces, and long-time defect dynamics. While these foundational studies are indispensable, the existing literature remains highly fragmented. A mechanism-centered synthesis is, therefore, required to bridge primary defect generation, material-specific extended-defect evolution, and their resulting mechanical responses. To address this gap, the present review is organized around a continuous defect-life-cycle framework: threshold displacement and Frenkel-pair production, cluster and extended-defect evolution, mitigation through chemical short-range-order-controlled defect energetics and grain-boundary-mediated annihilation, and finally dislocation/crack interactions and mechanical response.

After decades of efforts, atomistic simulation has gradually developed into an efficient research method in the field of radiation damage research, providing a more accurate basis for exploring the radiation damage mechanism and predicting the performance of materials under an irradiation environment. Table 1 compares the applicability, accessible time and length scales, principal advantages, limitations, and relative computational costs of mainstream computational methods for irradiation-damage simulations. These methods are complementary rather than mutually interchangeable. DFT and AIMD provide accurate defect energetics and migration barriers but are restricted by their high computational cost and limited system sizes. Classical MD explicitly resolves displacement cascades, Frenkel-pair recombination, and early defect clustering, whereas accelerated-dynamics methods extend the accessible time for selected thermally activated events. MLP-MD improves atomistic fidelity, particularly for chemically complex alloys, but remains dependent on representative training data and does not intrinsically overcome the MD timescale limitation. SRIM/TRIM efficiently predicts ion-transport and damage-production profiles without resolving explicit post-cascade defect evolution. At longer and larger scales, OKMC and cluster dynamics/rate theory describe defect kinetics and population evolution, while phase-field and DDD approaches address mesoscale microstructural evolution and mechanical response. Consequently, method selection should be based on the target physical process, required accuracy, accessible scales, and computational cost, and reliable irradiation-damage predictions generally require multiscale integration.

**Figure 1:**
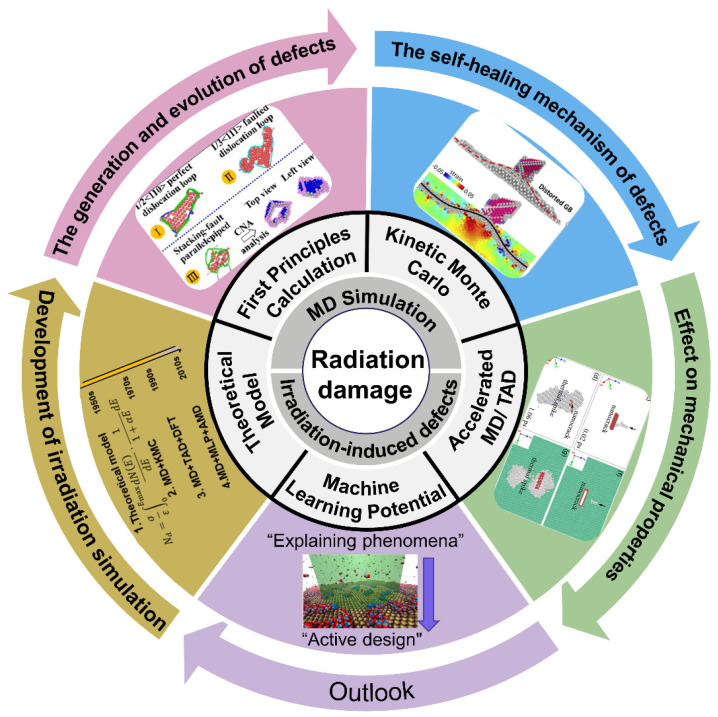
Schematic illustration of the advancement and application overview of computational simulations in the field of radiation damage.

Building on the abundant atomic-scale achievements that researchers have made in the field of irradiation damage, we have summarized these atomistic simulation findings, which not only facilitate understanding of irradiation damage mechanisms but also provide further guidance for the development of materials with excellent irradiation resistance. This review aims to summarize the development process and recent research hotspots of atomic simulations in the field of irradiation and highlight their typical applications in the research of defect evolution and irradiation performance, with schematic diagrams shown in Figure 1. Section 1 outlines the development and complementary roles of DFT, conventional and machine learning potential-based MD, SRIM, DDD, and mesoscale approaches, while retaining MD as the principal atomistic tool for resolving displacement-cascade dynamics. Section 2 describes the production of irradiation-induced point defects during the cascade collision process, summarizing the influence of various factors on the threshold displacement energy and the generation of Frenkel pairs. Section 3 examines the evolution of radiation-induced defects, with a focus on dislocation loops, stacking fault tetrahedra, voids, and helium bubbles. Section 4 summarizes the self-healing mechanisms of irradiation-induced defects in nuclear structural materials, highlighting the pivotal roles of short-range order and grain boundaries in mitigating radiation damage. Section 5 elaborates in detail on the effects of irradiation damage on the mechanical properties of nuclear structural materials. As the concluding section, Section 6 points out the limitations of current computational simulations in irradiation damage research of nuclear structural materials and outlines potential future research directions.

## 2. Irradiation-Induced Point Defects

The case studies selected in this section are organized according to both material class and nuclear relevance, rather than being intended as a direct ranking of irradiation tolerance. Cu, Mo, and Mg are discussed to elucidate the dependence of irradiation response on crystal structure, orientation, and temperature. Pure Fe is used as a BCC reference system for Fe-based ferritic/martensitic structural alloys, while Fe–Cr–Al alloys represent accident-tolerant fuel-cladding candidates. Fe–Ni–Cr/Ni-based alloys, including alloy 800H, are considered as FCC model systems for high-temperature reactor structural components. W and W–Ta alloys represent refractory BCC materials relevant to fusion plasma-facing applications. Furthermore, high-entropy alloys (HEAs, e.g., FeNiCrCoCu and AlxCoCrFeNi alloys) are included to investigate the impact of chemical complexity and compositional variations on irradiation damage.

### 2.1. Threshold Displacement Energy

The threshold displacement energy (TDE, *E_d_*) is the most fundamental quantity for describing material damage, and it is defined as the minimum energy required to move an atom from its lattice site to form a stable Frenkel pair (a vacancies–interstitial pair [49]) [50,51], which is a key parameter for evaluating the number of defects generated in the irradiation simulation process [52]. The formation of FPs requires overcoming the interatomic bonding forces within the crystal and the interactions between adjacent atoms, thereby resulting in the formation of a combination of a vacancy and an interstitial atom. In this process, the TDE value is roughly a factor of 5–10 higher than the FP formation energies. In simulating radiation damage in materials, such as the computer simulation package Stopping and Range of Ions in Matter (SRIM) [53], it is necessary to know the TDE of atoms in the material. Notably, the NRT model proposed by Norgett, Robinson, and Torrens has effectively addressed the difficulty in quantifying the TDE value. It can accurately calculate the atomic displacement damage of materials during irradiation. The NRT model predicted the number of atomic displacements (*N_d_*) as a function of cascade energy and is given by Equation (2) [19]:

(2) $$N_{d}\left(T_{d}\right)=\left[\begin{array}{ccc} 0 & & T_{d}<E_{d}\\ 1 & & E_{d}<T_{d}<\frac{2E_{d}}{0.8}\\ \frac{0.8T_{d}}{2E_{d}} & & \frac{2E_{d}}{0.8}<T_{d}<\infty \end{array}\right]$$

where the calculation results of the NRT model are very sensitive to the selection of TDE, and the number of defects generated by the damage energy (*T_d_*) is determined by TDE. In practical applications, it is necessary to accurately determine the value of TDE (basically below 100 eV [54]) to improve the accuracy of NRT model calculations.

By accurately calculating the change in TDE under different conditions, the model parameters for predicting radiation damage can be optimized, and the accuracy of prediction can be improved. The value of TDE is greatly influenced by crystal structure, which is attributed to the fact that the symmetry of crystal structure and the arrangement of atoms directly affect the interaction force and binding energy between atoms [55,56]. In general, the smaller the interatomic binding energy, the lower the TDE, which means that within the same crystal structure, the TDE varies among different crystalline orientations. In 1975, Kenik et al. [57] used a high-pressure electron microscope to study the variation in TDE of Cu and V with crystal orientation and found that the minimum TDE value of the Cu structure was <110>. However, not all nuclear structural materials are sensitive to crystallographic orientation, and as reported in [58], the TDE values in Mg exhibit weak directional dependence. Consequently, the averaging directional TDE values obscures critical structural vulnerabilities. Capturing the true resistance boundaries of the crystal lattice requires identifying maximum threshold values across specific crystallographic directions rather than relying on an isotropic mean.

**Figure 2:**
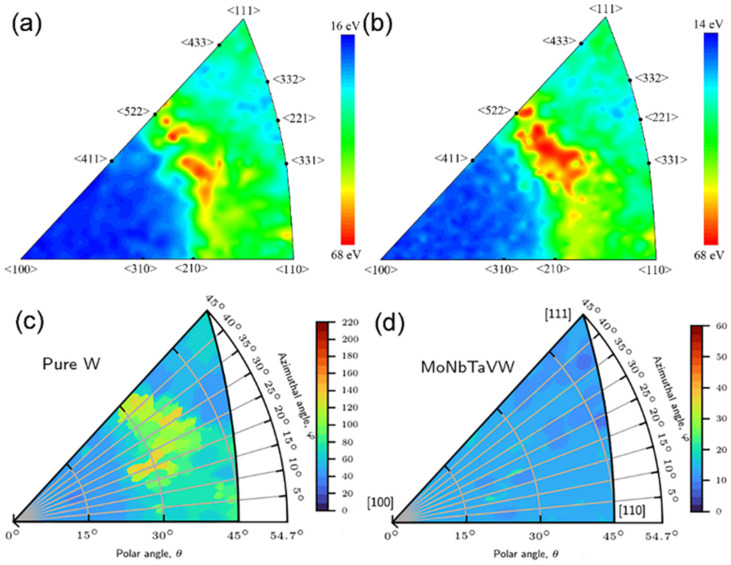
Fe provides a BCC reference for structural alloys, while W and MoNbTaVW elucidate chemical-component effects in fusion-relevant refractory BCC systems. Contour plot of the TDE surface for Fe as a function of crystal direction [64] using (**a**) the EAM potential [59] and (**b**) the F–S potential [65]. Angular maps of the TDE in the (**c**) pure W and (**d**) MoNbTaVW high-entropy alloys [61]. Reproduced with permission from Refs. [61,64].

The type of interatomic potential selected in calculations and the elemental arrangement and distribution in the alloy are key factors influencing the TDE value. Mendelev et al. [59] employed two types of interatomic potentials to determine the TDE value of Fe, and the results show that compared with the EAM potential, the TDE contour map of Fe calculated using the Finn–Sinclair potential exhibits multiple peak thresholds in the central region (in Figure 2a,b), which indicates that the interatomic potential exerts a non-negligible influence on the TDE value. Even though a variety of interatomic potentials exist currently, the EAM potential is still widely adopted to calculate the TDE of nuclear structural materials [20,60]. Byggmästar et al. [61] calculated TDE of pure W and MoNbTaVW high-entropy alloy (HEA) with random element arrangement by combining MLP with MD, as shown in Figure 2c,d. They found that the angular distribution map of the TDE of the high-entropy alloy is not only more uniform but also lower in value, which indicates that the anisotropy of TDE is significantly weakened by the random element arrangement [62,63,64].

The reliability of an interatomic potential for irradiation simulations depends not only on its equilibrium properties but also on its description of short-range repulsion, defect energetics, liquid configurations, and cascade defects. The TDE values in Table 2 demonstrate that the potential formalism alone does not determine the reliability of an irradiation simulation. Both the fitted database and the short-range connection to the repulsive potential are decisive. For example, under comparable simulation conditions for fcc Al, the orientation-averaged TDEs predicted using EAM–ZBL, MEAM–ZBL, and DP–ZBL are 16.73, 22.67, and 26.54 eV, respectively, compared with the recommended value of approximately 25 eV. Similarly, for bcc Mo, Ta, and W, tabGAP predicts minimum TDEs of approximately 31, 35, and 43 eV, whereas the corresponding EAM predictions are only 17, 21, and 25 eV. The tabGAP values are substantially closer to the available experimental estimates. In chemically complex alloys, a single scalar TDE is even less representative: the orientation-averaged TDE of MoNbTaVW changes from 44.3 eV in a random solid solution to 48.6 eV after the introduction of chemical short-range order. These comparisons indicate that irradiation potentials should be validated against directional and orientation-averaged TDEs, point-defect energetics, short-range repulsion, and cascade-defect statistics rather than equilibrium properties alone. Systematic comparisons show that no single potential is universally reliable. In Fe, different potentials produce distinct directional TDEs and defect-clustering fractions, whereas in W, the intermediate-distance connection between the equilibrium potential and the ZBL repulsive core strongly affects cascade morphology and surviving defect numbers. Machine-learned potentials improve the description of complex defect and liquid configurations but remain dependent on the completeness of their training data. Therefore, potentials should be selected and validated according to the specific target observable rather than their formalism alone.

In addition, TDE values are also affected by external factors such as temperature, primary knock-on atom (PKA) energy, strain, and the type of PKA atoms. Jung et al. [77] found that the TDE of Cu decreases from approximately 20 eV at 0 K to about 13 eV at 600 K. They proposed Equations (3) and (4) to explain the temperature dependence of the TDE value and attributed it to lattice softening and thermal vibration. The temperature dependence equation for TDE value is given by

(3) $$T_{d}\left(Q\right)=T_{d}\left(0\right)-△T_{vib}\left(Q\right)$$

(4) $$△T_{vib}\left(Q\right)=\frac{1}{2}K_{B}T\left(\begin{array}{c} \frac{f\left(\theta \right)}{mw^{2}} \end{array}\right)$$

where *T_d_* (0) is the TDE value at low temperature, and Δ*T_vib_* (*Q*) is the contribution of thermal vibration to the TDE value. *K_B_* is the Boltzmann constant, *T* is the temperature, *f* (*θ*) is a function related to the wavelength of lattice vibration, m is the atomic mass, and ω is the lattice vibration frequency. Through simulation calculations on several typical pure metals (Mo [78], Cu [77], and Fe [79]), it is found that the values of TDE generally show a decreasing trend with increasing temperature. In Fe–Cr–Al alloys, the temperature dependence of TDE is further modified by solute chemistry. Ye et al. [64] reported that the differences in TDE and directional dependence between Fe and Fe–Cr–Al are pronounced at low temperatures but become less distinct at elevated temperatures, which was attributed to an increased recombination radius and weakened solute-specific effects. Benjamin et al. [79] investigated the effect of strain on the TDE of Fe at 300 K and 500 K using MD simulation. The results show that the 5% strain has no clear regularity in its influence on TDE, because the effect of strain is non-uniform across different lattice directions, which may lead to a decrease in TDE along some directions while an increase in TDE along others. Subsequently, in the studies of Zheng et al. [10], the type of PKA atoms was further added to the influencing factors. They discovered that the anisotropy along the <110> and <135> directions strongly depends on the atomic type, with the degree of anisotropy of Fe being the highest, while that of Al is the lowest, which is closely related to atomic mass.

### 2.2. Frenkel Pairs and Clusters

**Figure 3:**
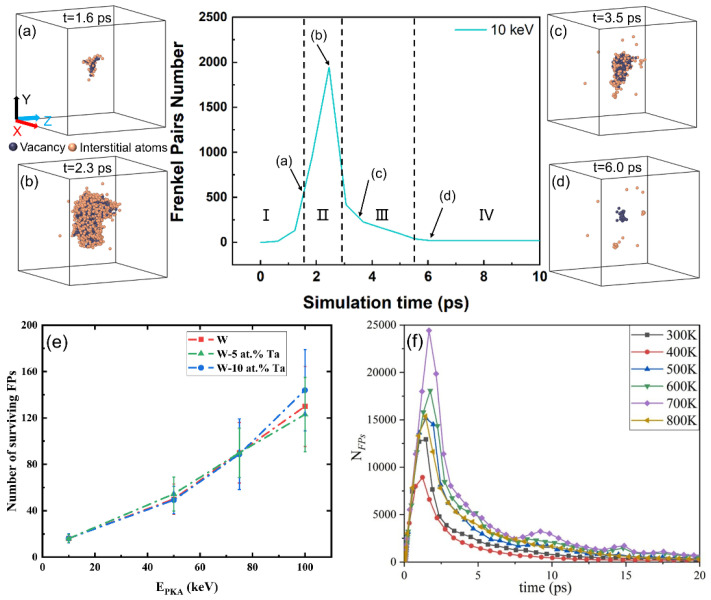
The Fe–Ni–Cr, W/W–Ta, and Fe–Cr–Al cases represent FCC high-temperature structural alloys, refractory fusion-facing materials, and accident-tolerant cladding candidates, respectively. (**a**–**d**) The variation and distribution of point defects in the collision cascade induced by 10 keV in Fe-Ni-Cr alloy over time was obtained through the Wigner–Seitz method. (**e**) The number of surviving FPs as a function of *E_PKA_*. Each data point is an average of 30 simulations, and the error bar is the corresponding standard deviation [84]. (**f**) Number of FPs as a function of time for 50 keV cascade damage at different temperatures [10]. Reproduced with permission from Refs. [10,84].

Surviving Frenkel pairs and their clusters, rather than the transient maximum defect population during the thermal spike, provide the principal link between primary displacement cascades and subsequent irradiation hardening, swelling, and embrittlement. It is, therefore, necessary to distinguish the peak Frenkel-pair population (N_peak_) from the surviving population measured after cascade cooling (N_sur_). Based on the statistics of existing simulation results, it is generally possible to divide the cascade collision process under irradiation into four stages, namely, (I) primary collision stage, (II) thermal spike stage, (III) initial collision residual stage, and (IV) defect migration stage [80,81,82]. For a more intuitive illustration of these four stages, we have plotted the time-dependent defect evolution curves of the Fe-Ni-Cr alloy at a PKA energy (*E_PKA_*) of 10 keV, as shown in Figure 3a–d. In the primary collision stage, PKA atoms induce supersonic displacement of lattice atoms through high-velocity collisions, and the FP count increases rapidly. Then, the number of FPs increased rapidly and reached the thermal spike stage in a very short period of time. Immediately afterward, the system enters the recombination stage, during which the rate of defect recombination accelerates, and the number of defects drops sharply. In the fourth stage, the evolution of displacement cascades is essentially completed, and long-range diffusion of defects occurs in this stage [83]. The *E_PKA_* is one of the key factors influencing the formation of FPs during irradiation. Qiu et al. [84] investigated the effect of *E_PKA_* on the generation and evolution of point defects in pure W, W-5 at.% Ta and W-10 at.% Ta alloys. The results show that increasing the cascade energy from 10 keV to 100 keV significantly enhanced defect formation in all alloys (Figure 3e). In the cascade collision process, the increase in the number of defects with increasing *E_PKA_* has been widely confirmed in various nuclear structural materials, such as W [85], Cu–Ni alloys [86], Ni–Fe alloys [87], FeNiCrCoCu alloys [88], etc. In addition, temperature is also an important factor affecting the formation of FPs, and the influence of temperature on FPs exhibits distinct behaviors across different alloy systems. As illustrated in Figure 3f, Zheng et al. [10] calculated the defect generation in Fe-Cr-Al alloys at different temperatures. The results indicate that temperature exerts a certain influence on the irradiation-induced defects in the Fe-Cr-Al alloy. While the peak defect concentration does not exhibit a clear linear relationship with temperature, the number of defects in the migration stage increases with rising temperature. Differently, He et al. [89] found that in the Ti6Al4V alloy, with increasing temperature, the peak number of FPs increases, while the number of surviving FPs during the defect migration stage decreases relatively. Zheng et al. [90] utilized MD simulations to investigate the irradiation process of W within the temperature range of 300–1500 K, and the results indicated that defect generation exhibits a weak correlation with temperature. The temperature-induced defect differentiation is primarily due to differences in energy barriers within the alloy. Meanwhile, the addition of different elements can alter the migration energies of vacancies and interstitial atoms in the alloy, thereby influencing defect recombination. Inconsistent temperature dependences across alloys can be reconciled by distinguishing cascade stages: higher temperatures increase the peak defect population (N_peak_) via thermal disorder but decrease the surviving population (N_sur_) by accelerating recombination. This competition explains the weak or non-monotonic trends observed in Fe–Cr–Al and W. Consequently, meaningful cross-study comparisons demand strictly consistent simulation parameters (e.g., PKA conditions, potentials, and defect-evaluation criteria).

**Figure 4:**
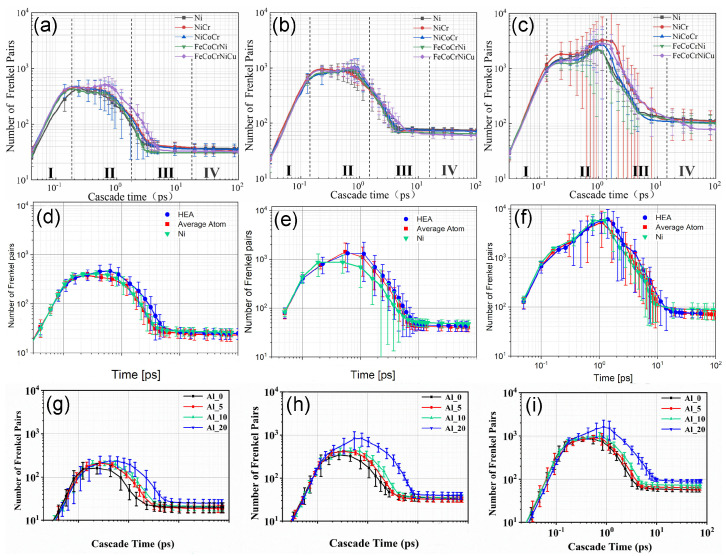
Variation in the FPs with cascade time. (**a**–**c**) The FeCoCrNiCu alloy at 10 keV, 20 keV, and 30 keV, respectively [91]. (**d**–**f**) FeNiCrCoCu alloy, average atom, and pure Ni at 10 keV, 20 keV, and 30 keV, respectively [94]. (**g**–**i**) AlxCoCrFeNi alloy with different Al contents at 5 keV, 10 keV, and 20 keV, respectively [83]. Comparison between FCC Ni and FeCoCrNi-based concentrated alloys. Reproduced with permission from Refs. [83,91,94].

At present, many studies on improving the radiation resistance of alloys are achieved by increasing the elemental complexity of alloys [91,92,93], which indicates that the number of element types within the alloy affects the generation of FPs. As shown in Figure 4a–c, He et al. [91] investigated the displacement cascade processes in Ni, NiCr, NiCoCr, FeCoCrNi, and FeCoCrNiCu HEAs. They found that under the same *E_PKA_*, the number of FPs in pure Ni always reaches the maximum first. With the increase in the compositional complexity of the alloy system, the time for the alloy to attain the maximum value is gradually delayed, and the number of FPs during the defect migration stage gradually decreases. However, increasing the complexity of the alloying elements does not necessarily enhance the radiation resistance of the alloy, as depicted in Figure 4d–f. Deluigi et al. [94] further verified this viewpoint by establishing an average atomic model. They averaged the properties of each constituent element in the high-entropy alloy and obtained a virtual material with average properties. The results showed that the FPs generation and evolution processes of high-entropy alloys and the average atom model were almost identical in the primary damage stage, indicating that the chemical complexity of high-entropy alloys itself is not the only condition for ensuring their radiation resistance. The content of alloying elements also exerts a certain influence on the FPs generated during the irradiation process. Wang et al. [83] studied the evolution of displacement cascades in AlxCoCrFeNi HEAs with different Al contents by MD simulation. Moreover, the influence of different Al contents on the number of residual defects shows a competitive effect. At low *E_PKA_*, the number of residual defects decreases first and then increases with the increase in Al content (Figure 4g,h), while at higher *E_PKA_* (such as 20 keV), the number of defects increases monotonously with the content of Al (Figure 4i). This highlights that the number of FPs generated during the alloy’s irradiation process can be controlled via the rational regulation of elemental content, thereby achieving radiation resistance. Taken together, these studies do not support a universal inverse relationship between chemical complexity and Frenkel-pair production. During the early ballistic stage, defect production is governed largely by average atomic mass, atomic density, elastic response, and cascade-energy partitioning, which explains why the average-atom and chemically complex models can exhibit similar primary damage. Local chemical disorder becomes more influential during cascade cooling by broadening the distributions of TDEs and migration barriers, trapping mobile defects, and modifying recombination and clustering. Chemical complexity may, therefore, delay the defect peak or reduce the surviving and clustered populations without necessarily decreasing the initial N_peak_. The energy-dependent effect of Al content further reflects competition between modified displacement thresholds and cascade density on the one hand, and defect trapping and recombination on the other.

In addition to increasing element types and regulating elemental content, there are also other common factors that affect the generation of FPs under irradiation. One approach is to introduce interstitial atoms [95,96], the mechanism of which lies in that interstitial atoms can increase the local lattice distortion of the alloy and inhibit vacancy diffusion, thereby enhancing the recombination of irradiation-induced point defects [97]. Wei et al. [98] reported that the mechanism by which interstitial atoms affect radiation resistance is strongly influenced by atomic size, and the smallest atoms play a decisive role. Another approach is to regulate the number and clustering behavior of FPs via applying strain. The conclusion derived from atomic simulations holds that tensile strain tends to increase the generation of FPs during the alloy’s irradiation [99,100,101], whereas compressive strain tends to reduce it [102,103]. The strain effect should not, however, be regarded as universal without specifying the strain state and PKA direction. Hydrostatic tension generally increases the available free volume and lowers some directional displacement barriers, whereas compression often increases these barriers. Differences among published results may, therefore, also originate from the imposed strain tensor, PKA direction, interatomic potential, and the time at which surviving defects are counted.

**Figure 5:**
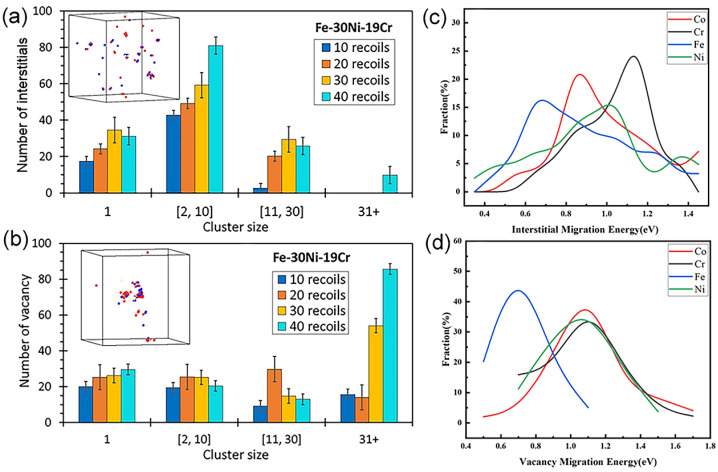
Alloy 800H (Fe–30Ni–19Cr) models an FCC high-temperature reactor alloy, whereas NiCoFeCr serves as a complex benchmark to assess migration-energy-controlled cluster evolution. (**a**) Interstitial clusters and (**b**) vacancy clusters, generated by cascade overlap in Fe-30Ni-19Cr [106]. Probability distribution of migration energy in NiCoFeCr homogeneous SP-CSAs: (**c**) interstitial migration energy and (**d**) vacancy migration energy [112]. Reproduced with permission from Refs. [106,112].

Clusters are the result of further aggregation and evolution of FPs under certain conditions, which can promote the formation and accumulation of more defects and exacerbate the degree of radiation damage to the material [104,105]. The smaller the size of defect clusters formed in the alloy after irradiation, the more it generally favors inhibiting the accumulation and growth of irradiation-induced point defects, thereby exhibiting superior radiation resistance. Cheik et al. [106] investigated the relationship between the size and number of clusters, and the number of overlapping cascades in alloy 800 H. They found that the number of clusters increases with the number of overlapping cascades, where small-sized clusters predominate, and the number of large-sized vacancy clusters is consistently greater than that of interstitial clusters (Figure 5a,b). This phenomenon also occurs in the irradiation cascades of V, V-5Ti-5Ta, and V-Ti-Ta alloys [107,108]. Differently, Qiu [84] and Lin [109] reported that the number of large-sized interstitial clusters is greater than that of large-sized vacancy clusters in W-Ta alloys and Ni-Co-Cr-Fe alloys under irradiation cascades. Vacancy and interstitial clustering biases stem from a complex interplay of defect energetics, cascade dynamics, and irradiation conditions, rather than migration barriers alone. Furthermore, methodological choices—such as interatomic potentials and identification criteria—heavily influence defect classification [110,111]. Deng et al. [112] calculated the migration energy barrier spectra of different types of interstitial atoms and vacancies, and the results indicated that Fe atoms possess relatively low interstitial migration energy (Figure 5a) and relatively high vacancy migration energy (Figure 5b), which facilitates the formation of larger-sized interstitial clusters.

Evaluating primary damage requires a hierarchical, complementary modeling strategy. DFT provides foundational defect energetics but is restricted by spatio-temporal limits. Classical MD captures large-scale cascade dynamics yet depends heavily on empirical potentials. MLPs bridge this gap, offering DFT-level accuracy at MD scales, though limited by training data dependency and high-energy extrapolation risks. Ultimately, these methods represent complementary levels of fidelity rather than interchangeable tools. Overall, three common trends emerge from the available studies. First, the absolute peak and surviving Frenkel-pair populations generally increase with PKA energy, whereas the surviving-defect efficiency normalized by cascade energy does not necessarily increase because in-cascade recombination and sub-cascade formation also become more pronounced. Second, temperature, alloy chemistry, and strain generally exert a stronger and more system-dependent influence on N_sur_ than on the transient N_peak_. Third, chemical complexity more consistently broadens defect-energy and migration-barrier distributions and suppresses defect mobility or large-cluster formation than it suppresses the initial production of Frenkel pairs. The discrepancies among published simulations can, therefore, be divided into physical sources, including crystal structure, alloy chemistry, PKA energy and direction, temperature, strain, and defect sinks, and methodological sources, such as the interatomic potential, short-range repulsion, electronic stopping, boundary conditions, cell size, statistical sampling, defect definition, cluster criterion, and observation time. Explicitly reporting and controlling these variables is essential for meaningful cross-study comparisons.

## 3. Evolution of Irradiation-Induced Defects

Building on the material-selection framework established in Table 3 and Section 2, this section focuses on the material-dependent evolution of primary irradiation defects into extended structures. The preferred evolution pathway from point defects to dislocation loops, stacking-fault tetrahedra, voids, or gas bubbles is governed by the interplay among crystal structure, defect migration kinetics, stacking-fault energy, vacancy–gas interactions, and microstructural sinks. Accordingly, the following subsections compare the evolution pathways of the representative material systems introduced above and clarify the material context of each selected example.

### 3.1. Dislocation Loop

With increasing irradiation dose and duration, point defects undergo aggregation and migration, and this process of irradiation damage accumulation leads to the formation of dislocation loops. The dislocation loop can induce additional stress concentration regions within the material, and these regions tend to act as crack initiation sites, thereby deteriorating the material’s mechanical properties [113,114]. Hence, an understanding of the evolution processes and underlying mechanisms from point defects to dislocation loops is crucial to the study of irradiation damage mechanisms.

**Figure 6:**
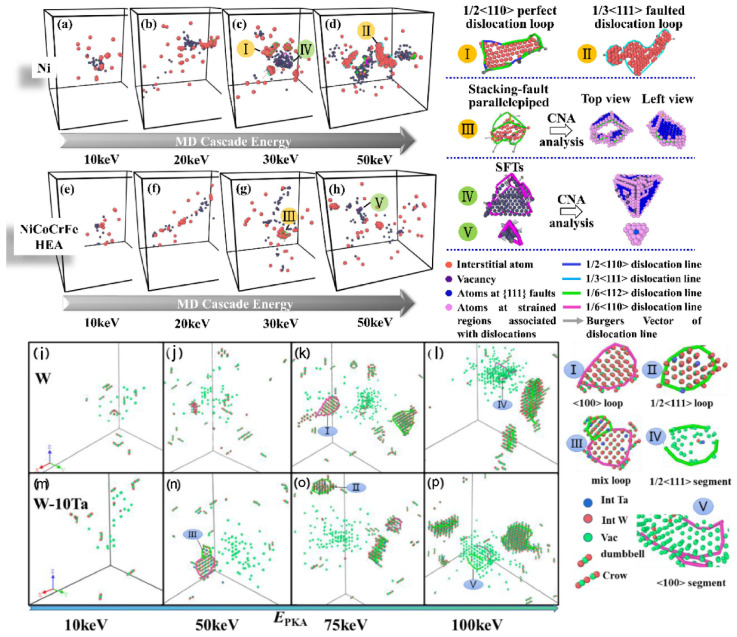
Ni and NiCoCrFe serve as baseline and complex FCC models, respectively, while W and W–Ta exemplify fusion-relevant refractory BCC alloys. Snapshots of defect distributions at the end of displacement cascades as a function of *E_PKA_*: (**a**–**d**) Ni at 10 keV, 20 keV, 30 keV, and 50 keV, respectively. (**e**–**h**) NiCoCrFe HEA at 10 keV, 20 keV, 30 keV, and 50 keV, respectively. Detailed views of dislocation loops (**I**,**II**), stacking-fault parallelepiped (**III**), and SFTs (**IV**,**V**) are provided [109]. (**i**–**l**) W at 10 keV, 50 keV, 75 keV, and 100 keV, respectively. (**m**–**p**) W-10 at.% Ta alloy at 10 keV, 50 keV, 75 keV, and 100 keV, respectively. Detailed views of dislocation loops (**I**–**V**) are provided [84]. Reproduced with permission from Refs. [84,109].

The *E_PKA_* can directly affect the evolution process and formation mechanism of dislocation loops during the displacement cascade process. At low *E_PKA_*, dislocation loops are formed by absorbing high-mobility point defects and defect clusters. Lin [109] and Qiu [84] have respectively conducted detailed atomistic simulations on the formation of dislocation loops in NiCoCrFe HEA and W-Ta alloys during irradiation. At 10 keV, the defects in Ni, NiCoCrFe HEA, W, and W-Ta alloys are predominantly vacancies and interstitial atoms, as shown in Figure 6a,e,i,m. When *E_PKA_* increased to 20 keV, small interstitial atomic clusters appeared, indicating that individual interstitial atoms began to accumulate and form clusters (Figure 6b,f). With the further increase in the *E_PKA_* to 30 keV, interstitial clusters first emerged and began to form 1/3<111> dislocation loops in NiCoCrFe HEA (Figure 6g). In other MD simulation studies on Ni-based alloys [115,116,117,118], it can also be observed that 1/2<110> dislocation loops and 1/3<111> dislocation loops are present. Due to differences in migration energies, W-Ta alloys only start to form 1/2<111> dislocation loops at 50 keV (Figure 6n). It should be noted that atomic simulation studies have reported that all complete dislocation loops formed during alloy irradiation are predominantly interstitial dislocation loops [101,119,120], which may be accompanied by 1/2<100> vacancy dislocation loops during high-energy irradiation (Figure 6k,o).

At high *E_PKA_*, the formation mechanism of dislocation loops is mainly dominated by the coalescence of two or more dislocation loops of different sizes. Under high irradiation energy, the dislocation loop density within the alloy gradually saturates, followed by the formation of amorphous regions, with the most distinct characteristic being the coalescence of dislocation loops into ultra-large ones (as shown in Figure 6l,p). Liu et al. [40] developed a W model via MLP training, and found that under 200 keV irradiation, the majority of dislocation loops in W are of the 1/2<111> type, accompanied by dislocation loop coalescence. Fu et al. [121] irradiated the W-Re alloy with an ultrahigh energy of 300 keV, and during the process, they observed that two 1/2<111> loops migrated and coalesced to form a large mixed loop. Han et al. [122] found that at a relatively high irradiation dose of 0.03 dpa, the prismatic dislocation loops in the FeCoNiCrCu HEA are gradually absorbed by Frank dislocation loops during the cascade process, forming larger Frank dislocation loops.

Notably, the dislocation loop can further evolve into stacking fault tetrahedra (SFTs) after their formation. Most atomic simulations have demonstrated that dislocation loops play a crucial role in the formation of SFTs during irradiation [6,123,124]. Studies have demonstrated that the core of the displacement cascade retains highly vacancy-enriched dislocation loops, which subsequently irreversibly transform into SFTs to minimize the total free energy of the system [125]. Ding et al. [126] discovered via MD simulations that <100> dislocation loops in W can be transformed into 1/2<111> vacancy-type dislocation loops through interloop reactions under 30 keV irradiation, providing a structural basis for the transition of vacancy clusters to SFTs. It has been well established that supersaturated vacancies aggregate on the low-energy {111} planes to form planar vacancy clusters. Once these vacancy clusters grow to a critical size, the corresponding atomic plane undergoes collapse, forming a triangular Frank partial dislocation loop (Frank loop) enclosing a stacking fault, with a Burgers vector of a/3<111> [127]. Triangular Frank loops in low stacking fault energy systems are energetically unstable and spontaneously dissociate along their three edges into Shockley partial dislocations. It is observed in Figure 6c that the collapse of aggregated voids forms an SFT composed of six 1/6<110> stair-rod dislocation loops. Wirth et al. [128] demonstrated the formation process of SFTs, which primarily involves the collapse of vacancies to form dislocation loops, followed by the slip of Shockley dislocations along {111} planes toward the vertices of the tetrahedron to form SFTs.

### 3.2. Voids and Bubbles

**Figure 7:**
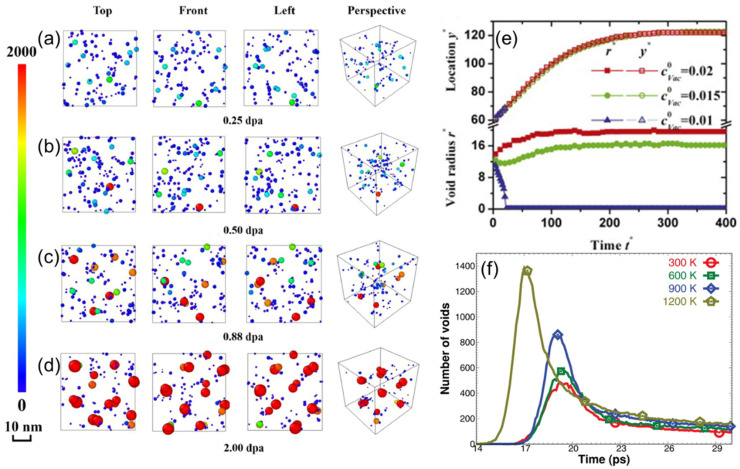
W panels illustrate void evolution in fusion-grade refractory metals, whereas Fe serves as a BCC baseline for temperature-driven void nucleation. Microstructure of irradiated W at a dose of (**a**) 0.25 dpa, (**b**) 0.5 dpa, (**c**) 0.88 dpa, and (**d**) 2 dpa, respectively. Each sphere represents a vacancy-type object [132]. (**e**) Effects of the initial vacancy concentration C_vac_ on the normalized void radius r* and the normalized y coordinate y* of the void center as functions of normalized time t*. r* characterizes void growth or shrinkage, whereas y* tracks void migration along the temperature-gradient direction [144]. (**f**) Overall number of voids as a function of time for single crystal iron at each applied temperature [136]. Reproduced with permission from Refs. [132,136,144].

With increasing irradiation dose, vacancy clusters tend to aggregate and evolve into stable voids [123]. Void nucleation is essentially a process of vacancy clustering, and this process is strongly dependent on irradiation energy [129,130,131]. Li et al. [132] investigated the void evolution behavior in W under different irradiation doses, reporting a sharp increase in the total number of vacancies at the initial irradiation stage (0–0.25 dpa) and the spontaneous formation of regular voids from vacancies with increasing irradiation dose (see Figure 7a–d). Increasing the void concentration (CVac = 0.015, 0.02) stabilizes pre-existing voids and promotes their further expansion (Figure 7e). Beyond that, Zeng et al. [133] simulated the evolution of the volume fraction (porosity), number, and diameter of voids in alloys with irradiation time, and classified the void formation process into three stages, namely nucleation, growth, and coarsening. In the nucleation stage, no voids form in the matrix owing to insufficient vacancies; both the number density and size of voids increase dramatically in the growth stage, and voids undergo continuous coarsening in the coarsening stage as all available nucleation sites in the matrix are completely exhausted. Rudd et al. [134] performed MD simulations on void growth in metals (V, Nb, Mo, Ta, and W) using the Finnis–Sinclair potential. They found that under the condition of low irradiation energy, the generated vacancies mainly exist in the form of single vacancies or small vacancy clusters, and void nucleation mainly relies on the long-range migration, encounter, and coalescence of single vacancies. Under high irradiation energy conditions, the direct formation of vacancy clusters by displacement cascades serves as the primary mechanism for void nucleation [135]. This is primarily attributed to the fact that high energy facilitates the rapid overcoming of the energy barrier for void nucleation, thereby enhancing the nucleation rate. This energetic nucleation acceleration underlines the necessity of multiscale modeling where continuum differential formulations have historically proven essential for describing late-stage cavities. Specifically, a landmark review by Nordlund highlighted that reaction rate equations remain highly effective for predicting the average concentration evolution during prolonged void and helium bubble growth [44].

Studies have shown that temperature can regulate the void formation process by influencing the vacancy diffusion rate and defect recombination rate, a process dominated by defect migration and dislocation dynamics. Rawat et al. [136] investigated the effect of temperature on void evolution in Fe; they revealed that the peak void number density is achieved at 1200 K compared with that at 300 K, a finding that demonstrates the significantly enhanced void nucleation behavior at elevated temperatures (in Figure 7f). At low temperatures, vacancy diffusion is limited, and vacancy nucleation is mainly based on the “site saturation” mechanism [133,137]. This process significantly impedes the mobility of vacancies, leading to the formation of a vacancy supersaturation state. At high temperatures, voids rapidly grow through the Ostwald ripening mechanism, and the critical nucleation size increases [133,138,139], attributed to enhanced vacancy diffusion and higher mobility of interstitial atoms at high temperatures. Additionally, the application of a stress field can significantly affect the growth of vacancy clusters, leading to the formation of voids [134,140,141,142]. Sali et al. [143] found that when the tensile strain was increased to 5%, and the *E_PKA_* remained constant (10 keV), the formation of voids could always be observed.

After void formation, the existing voids can exert a certain influence on the defects generated by subsequent cascade processes. Fellman et al. [145] investigated the effect of pre-existing voids in alloys on the generation of new defects during the cascade overlap process. They found that the number of new defects is logarithmically dependent on the size of pre-existing voids. Nnew depends on the size of the pre-existing voids according to Equation (5) [146].

(5) $$N_{new}=\{\begin{array}{c} N_{0}\;N_{vac}=0\\ N_{0}-a{\ln N}_{vac}\;N_{vac}>0 \end{array}$$

Here, *N*_0_ is the number of new defects in pristine material, and *N_vac_* describes the size of the pre-existing defect in terms of the number of vacancies it contains. a is the fitting parameter, which is influenced by the energy level of PKA ($a=b\sqrt{E_{PKA}}$, *b* = 0.1970 ± 0.0079).

**Figure 8:**
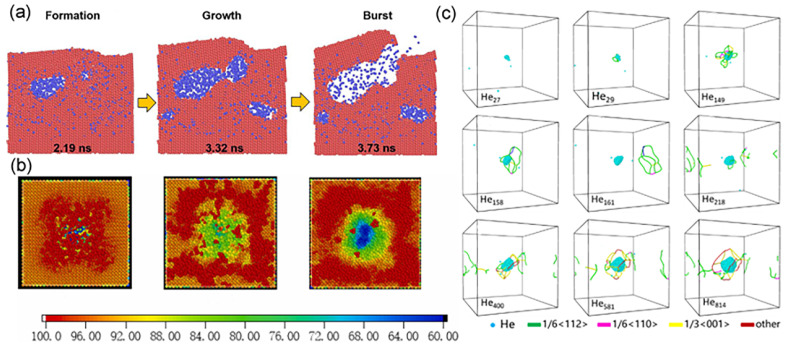
(**a**) Snapshots of evolution of bubbles below the (1 1 1) surface of W, exposed to the flux of 6.24 × 1028 m^−2^ s^−1^ and 80 eV at 300 K. The red spheres represent the W atoms, and the blue spheres represent the D atoms [150]. (**b**) Surface snapshots at 1800 K at different times. The color bar shows the height in the Z direction, with the simulation cell bottom at 0 a [155]. (**c**) The He bubble and the related microstructure evolution in nickel bulk at 900 K during bubble growth with a time interval of about 10 ps between sequential injections [156]. Gas-bubble behavior in BCC W and FCC Ni. Reproduced with permission from Refs. [150,155,156].

When insoluble gases are present, or there is a sufficiently large net vacancy flux, vacancy clusters are more likely to aggregate to form stable helium (He) bubbles. He bubbles can cause the material to become brittle, and the degree of brittleness depends on the formation mechanism of the bubbles during the irradiation process [147,148]. In the previous study, Kobayashi et al. [149] discovered the self-induced He bubble growth mechanism, in which He bubbles, once formed in W, absorb migrating He atoms and push surrounding W atoms to form a self-interstitial atom cloud, thereby promoting new bubble nucleation and growth. Xiang et al. [150] simulated the evolution process of D bubbles beneath the W (111) surface and found that, as the cumulative fluence increases from 2.5 × 10^19^ m^−2^ to 1.0 × 10^20^ m^−2^, small bubbles form. When two bubbles are large enough to come into contact with each other, they will merge to form a larger bubble (Figure 8a). Due to the enormous gas pressure inside, the largest bubble ruptures upon reaching the surface, creating a larger cavity in W. It is worth mentioning that Smirnov et al.’s [151] simulation of the coalescence of two bubbles revealed that the merging occurs through the lateral growth of the bubbles, rather than along the shortest connection length. During bubble formation, bubbles incorporate voids and grow in size [152], with the inhomogeneity in bubble distribution observed during this process attributed to the anisotropic stress fields formed around the bubbles [151,153,154]. Zhang et al. [155] further observed that the surface morphology of W changes drastically during bubble rupture. He bubbles rupture when the “humping” formed on the W surface rises approximately 1 nm above the original surface. Most W atoms ejected from the W matrix at the He bubble apex alongside He atom escape eventually return to the surface, as illustrated in Figure 8b. Simply put, the evolution process of bubbles in crystals can be roughly divided into three stages: formation, growth, and burst.

The process of He bubble formation is concomitantly associated with the generation of dislocations, among which the dominant type is Shockley dislocations. Wen et al. [156] investigated the evolution of dislocations during the growth of bubbles in Ni and found that the dislocation density increases with the enlargement of bubbles. Due to the low stacking fault energy (SFE), the initially formed dislocations are mainly of the Shockley type. Continuous dislocation multiplication occurs, and after the formation of prismatic dislocation loops, the dislocations are pushed out by bubbles and further separated along the <110> direction, thereby relieving pressure, as illustrated in Figure 8c. Once the bubble pressure is relieved, it is observed that new dislocations continue to form new Shockley partial dislocation loops around clusters. Cui et al. [152] reported that when bubbles are subjected to overpressure, it facilitates the formation of interstitial dislocation loops. In contrast, underpressure inside He bubbles tends to favor the generation of vacancy dislocation loops. This indicates that the evolution of dislocations is influenced by the state of bubbles.

The stability of bubbles exerts a significant impact on the generation of dislocations and the evolution of the microstructure [157,158], and it is determined by the ratio of their internal vacancies to He atoms. Studies have shown that when the Vac/He ratio is in the range of 0.5–1, the internal pressure of bubbles has not yet saturated, and the generation of dislocations is suppressed [152,159,160]. Pu et al. [161], through their research on high-energy radiation cascades impacting He bubbles, found that the stability of the bubbles depends on their vacancy/He atom ratio (Vac/He), with the relatively stable ratio being 1:1. Then, Neogi et al. [162] investigated the stability and strengthening effect of He bubbles in single-crystal copper at different concentrations of He atoms. They found that solid He was formed in helium bubbles when the Vac/He was 1:2 and 1:3. The formation of solid He can significantly improve the mechanical properties of irradiated metals, but this strengthening effect weakens when the He concentration exceeds a certain threshold (Vac/He ratio 1:3). Generally, when the Vac/He ratio is less than 1, the internal pressure of bubbles cannot be released through defects. When the Vac/He ratio is greater than 1, the bubble pressure reaches the saturation region, and defects are subsequently formed in the material, which is the main mechanism for bubble pressure release.

Taken together, the evolution of irradiation-induced extended defects is strongly material dependent. In W and W-based refractory materials, the coupled evolution of dislocation loops, vacancy clusters, and H/He bubbles is closely associated with fusion-facing conditions and surface morphology changes. In Fe-based BCC structural materials, vacancy migration, loop nucleation, and bubble-assisted defect accumulation govern swelling and embrittlement. In FCC Ni/Fe–Ni–Cr systems, faulted loops and stacking-fault tetrahedra are additionally influenced by stacking-fault energy, while compositionally complex alloys are used to determine how local chemical disorder alters loop and cluster stability. In HCP Zr alloys, anisotropic defect–dislocation interactions require separate consideration. Consequently, cross-scale comparisons should prioritize underlying mechanisms over direct quantitative equivalence. To clarify the material scope of this review, Table 3 summarizes the representative metallic materials frequently investigated in irradiation-damage simulations. Their typical applications, crystal structures, dominant irradiation-induced defect features, and selection rationale are listed to provide a material-specific context for the subsequent discussions of defect formation, evolution, and mechanical-property degradation. Although primary vacancies and self-interstitial atoms are produced in all irradiated metallic materials, the formation of extended defects shows clear material dependence. BCC Fe-based alloys commonly exhibit <111>/<100> dislocation loops and vacancy clusters, whereas FCC austenitic/Ni-based alloys frequently develop faulted/perfect loops, SFT, cavities, and radiation-induced segregation. W-based refractory materials are characterized by interstitial loops and, under hydrogen/helium irradiation, gas–vacancy complexes and bubbles, while HCP Zr alloys commonly show basal/prismatic loops and irradiation growth. In compositionally complex alloys, local chemical disorder further modifies defect migration, clustering, and stability.

MD provides the foundational post-cascade defect inventory and spatial correlations. Subsequently, KMC/OKMC extend timescales using kinetic parameters, though constrained by predefined event catalogs. Rate theory and cluster dynamics efficiently predict long-term, high-dose evolution, yet their mean-field nature sacrifices spatial details. Finally, phase-field modeling resolves complex mesoscale morphology and interface evolution, relying on robust thermo-kinetic inputs. As mentioned above, the formation mechanisms of dislocation loops can be roughly categorized into two types. One is through the absorption of the aforementioned high-mobility point defects and defect clusters, and the other is via the coalescence of two or more dislocation loops of different sizes. The nucleation and formation mechanism of irradiation-induced voids in alloys is dominantly governed by the defect migration energy barrier and dislocation dynamics, while the formation of He bubbles is not only controlled by the above factors but also critically regulated by the Vac/He ratio in the alloy. Looking forward, the primary challenge lies in developing multiscale predictive frameworks that can map the discrete atomistic phenomena of bubble coalescence and surface burst onto macroscale multiyear microstructural degradation models under high flux reactor conditions.

## 4. The Self-Healing Mechanism of Defects

### 4.1. Chemical Short-Range Order

**Figure 9:**
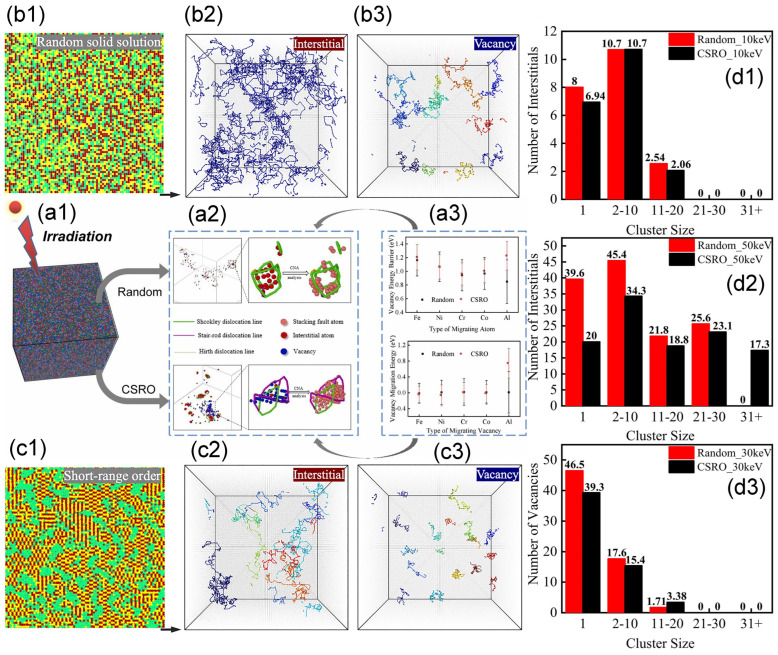
Effects of short-range order on defect evolution in medium- and high-entropy alloys: (**a1**) Model schematic diagram, (**a2**) distribution of defect clusters in the random alloy and CSRO alloy at 50 keV E_PKA_, and (**a3**) migration energy according to the migration atom type [167]. Short-range order traps point defects, reducing their diffusivity. Interstitial and vacancy diffusion trajectories in (**b1**–**b3**) a random solid solution of CrCoNi and (**c1**–**c3**) thermally annealed CrCoNi with chemical short-range order [168]. The size distribution of (**d1**) both interstitial atom clusters and vacancy clusters, (**d2**) interstitial atom clusters, and (**d3**) vacancy clusters [167]. Reproduced with permission from Refs. [167,168].

Internal defects in alloys are a key factor threatening their performance, so exploring the self-healing mechanisms of alloy defects is of great significance for improving radiation resistance. Chemical short-range ordering (CSRO) in alloys refers to a phenomenon in which atoms of specific elements in alloy solid solutions exhibit preferential distribution, deviating from a completely random distribution [163]. Existing studies have shown that the presence of CSRO contributes to the irradiation resistance of nuclear structural materials [164,165], primarily because it modifies the SFE of the alloy and the local energy barriers for point defects. Li et al. [166] demonstrated that the CSRO in CoNiCrFeMn HEA formed by annealing at 1100 K can more effectively retard the increase in the number of point defects, which indicates that it has better radiation resistance. Li et al. [167] investigated the effect of CSRO on the irradiation damage behavior of AlCoCrFeNi alloy via hybrid MD and KMC simulations. The calculation results show that the CSRO structure effectively inhibits the generation of point defects in AlCoCrFeNi alloy during irradiation, and this inhibitory effect strengthens with the increase in irradiation energy. They attribute the CSRO-enhanced radiation tolerance to the high formation energies of vacancies and interstitial atoms as well as the high vacancy migration energy barriers (Figure 9(a1–a3)). Similarly, Cao et al. [168] elucidated the role of CSRO in point defect diffusion by demonstrating the diffusion trajectories of interstitials and vacancies in random solid solutions (Figure 9(b1–b3)) and CoCrNi alloys with CSRO (Figure 9(c1–c3)), thereby confining and localizing defect diffusion. Directly related to radiation resistance is the size and number of clusters formed. As can be seen from Figure 9(d1–d3), under the same irradiation conditions, the number of small-sized clusters generated in CSRO alloy is significantly lower than that in random solid solutions, indicating that CSRO order exerts an inhibitory effect on clusters at radiation damage sites.

**Figure 10:**
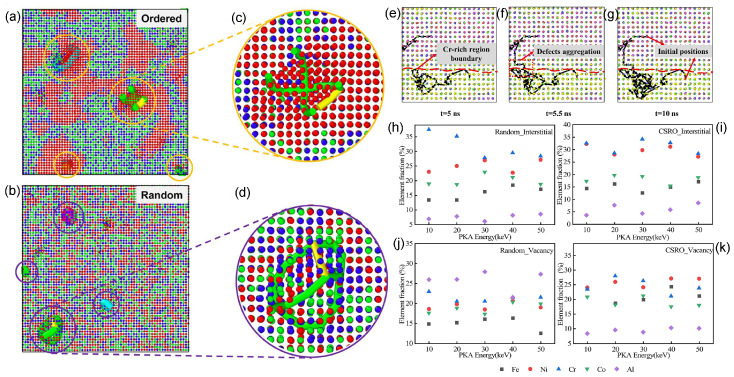
Compositionally complex FCC alloys reveal how chemical ordering alters loop formation and interstitial diffusion. Distribution of dislocation loops in (**a**) the ordered sample and (**b**) the random sample. Enlargements of the regions circled in (**a**,**b**) are shown in (**c**,**d**), respectively. Green, blue, and red balls represent Ni, Fe, and Cr atoms, respectively [164]. (**e**–**j**) Diffusion simulation of two interstitial defects with distinct initial positions in CoNiCrFeMn after annealing at 600 K. Black lines denote the diffusion trajectories of interstitials; red dashed lines denote the Cr-rich region boundary [166]. (**h**–**k**) The relationship between stable survival defects and elements with different *E_PKA_* [167]. Reproduced with permission from Refs. [164,166,167].

Theoretically, the presence of CRSO may promote the nucleation of dislocation loops, mainly because CRSO in the solid solution structure induces severe lattice distortion. Several studies have reported the mechanism underlying the effect of CRSO on the irradiation resistance of alloys from a structural perspective. Liu et al. [164] reported that in CRSO CrFeNi multi-principal element alloys (MPEAs), dislocation loops formed after radiation damage tend to preferentially nucleate in Cr-rich regions (Figure 10a,c), whereas no such preferential formation of dislocation loops was observed in random solid solution alloys (Figure 10b,d). Further investigating the defect evolution in Cr-enriched regions is crucial for clarifying the mechanism by which CRSO influences radiation effects. Through 10 ns diffusion simulations of two interstitial defects, Li et al. [166] observed rapid defect diffusion at the bottom region of the model. Internal stress induced by lattice distortion drives defects to coalesce and migrate to Cr-enriched regions after 5.5 ns (Figure 10e,j) and promotes dislocation loop aggregation by providing defect diffusion momentum. Another viewpoint holds that the presence of CRSO is mainly associated with Cr-Cr clusters in Cr-rich regions, in which the weak bonds between Cr atoms cause the local structure to tend to collapse, thus serving as nucleation sites for dislocation loops [169]. In addition, Li et al. [167] reported in their study on the Al_0_._5_CoCrFeNi MPEA system that the effects of CSRO on different types of defects exhibit significant differences. It hardly affects the elemental composition of interstitial atoms, but significantly regulates that of vacancies, endowing the formation of vacancies with element dependence. Compared with random solid solutions, vacancies in CRSO alloys exhibit a much lower content of Al (Figure 10h–k).

### 4.2. Grain Boundary

**Figure 11:**
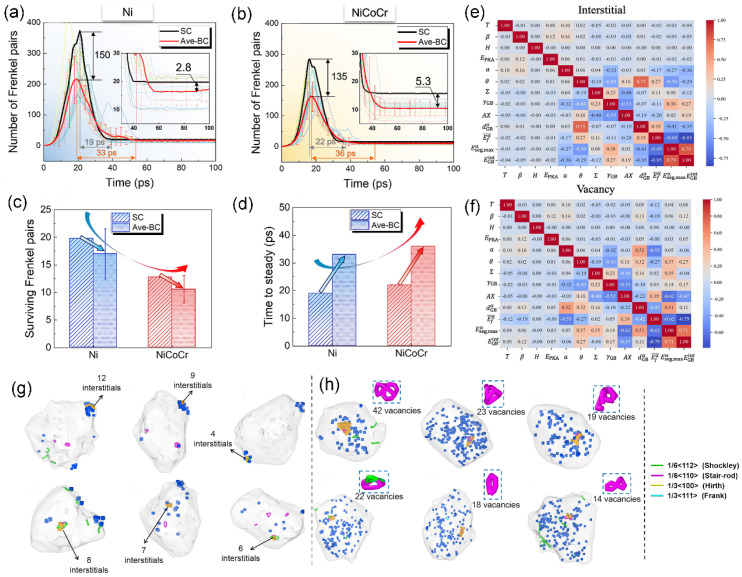
Ni, NiCoCr, and FeCoCrNi alloys reveal how alloying and GBs influence irradiation defect evolution. (**a**,**b**) Variation in the number of FPs inside the grain with the simulation time for Ni and NiCoCr. (**c**) The number of surviving FPs in the single-crystal (SC) and bi-crystal (BC) samples. (**d**) Total time taken for the cascade reaction to reach the stable stage from the peak stage [175]. (**e**,**f**) Pearson correlation matrix for the 13 composition-derived features. The color of each square reflects the correlation coefficient (r) between variables, where red represents positive correlation, blue represents negative correlation, and darker shades indicate stronger correlations [180]. Distribution profiles of defects and dislocation networks in three typical grains for (**a**–**c**) NC-Ni and (**d**–**f**) NC-HEA; (**g**) interstitial atoms; (**h**) vacancies [181]. Reproduced with permission from Refs. [175,180,181].

Nanocrystalline structural materials and nano-dispersed structural materials have garnered widespread attention due to their superior radiation damage tolerance compared to coarse-grained structural materials, with the key reason being the abundance of grain boundaries (GBs) in nanocrystals [170,171,172]. As a ubiquitous crystalline defect in alloys, GBs are generally regarded as the key to enhancing alloy properties. In the field of radiation damage, they have been proven to mitigate radiation damage by absorbing irradiation-induced defects [173,174], serving as an extremely important defect self-healing strategy. GBs function as efficient sinks for defects induced by irradiation, with atomic-scale evidence provided by Tan et al. [175] by MD. Cascade evolution results for pure Ni demonstrate that the peak population of FPs in a bi-crystal model is reduced by 40 to 45 percent compared to a single crystal. This reduction is accompanied by a significant decrease in steady-state residual defects, which directly confirms the persistent defect trapping effect of GBs during the cascade collision process (Figure 11a,b). Subsequent quantitative analysis of defect populations reveals that the introduction of GBs in the NiCoCr system results in an average reduction of 5.3 steady-state surviving FPs compared to the single-crystal counterpart (Figure 11c). This reduction is nearly twofold that observed in pure Ni, which exhibits a decrease of 2.8, providing direct evidence that the defect sink efficiency of grain boundaries in NiCoCr is significantly superior to that in pure Ni. Both the GB and the multi-principal alloying effect significantly prolong the relaxation duration from the defect peak to the steady state (Figure 11d). This result is consistent with those reported by Zhang et al. [176]. In addition, Parfitt et al. [157] confirmed that the presence of GBs in W prevents the recombination of interstitial atoms and vacancies during collision cascades, which causes vacancies to be more likely to form large clusters than interstitial atoms, and the temperature dependence of this process is not strong. Extensive research indicates that GBs exhibit a pronounced preference for interstitial absorption, which is primarily attributed to the lower migration energy and resultant higher diffusion rates of interstitial atoms [177,178,179].

Understanding the coupled influence of multidimensional factors on defect absorption is essential to deciphering GB mechanisms. By employing Pearson correlation analysis, Huang et al. [180] quantified the impact of 13 variables across four categories on the absorption rates of interstitials and vacancies in FCC systems. Their study reveals that severe multicollinearity exists among defect formation energy parameters for both interstitial (Figure 11e) and vacancy systems (Figure 11f). While defect formation energy parameters exhibit severe multicollinearity, the lattice constant, grain boundary energy, and defect GB interaction width are identified as the dominant features for both defect types. Notably, external irradiation conditions such as temperature and PKA parameters show negligible influence. These results statistically validate that the intrinsic attributes of GBs and materials dictate their defect absorption capacity. Consequently, this work establishes a robust physical foundation and a criterion for feature selection in subsequent data-driven modeling and prediction of GB performance. Distinct from idealized models with isolated GBs, engineering-grade polycrystalline materials feature three-dimensional interconnected grain boundary networks as their core structural characteristic. Liu et al. [85] have reported on irradiation in polycrystalline W systems, showing that the smaller the grain size, the greater the number of defects formed within GB regions. Results from Zhu et al. [181] confirm that these networks achieve comprehensive defect capture throughout the material volume (Figure 11g,h). Specifically, the distributed absorption of high mobility interstitials by the network prevents the development of large interstitial clusters typical of simpler systems. Furthermore, this exhaustive interstitial sequestration shifts the FP balance, which indirectly prevents vacancy cluster coarsening and lowers the incidence of large-scale vacancy defects. By moving beyond bi-crystal approximations, this research elucidates the synergistic mechanisms within polycrystalline networks during extended irradiation.

**Figure 12:**
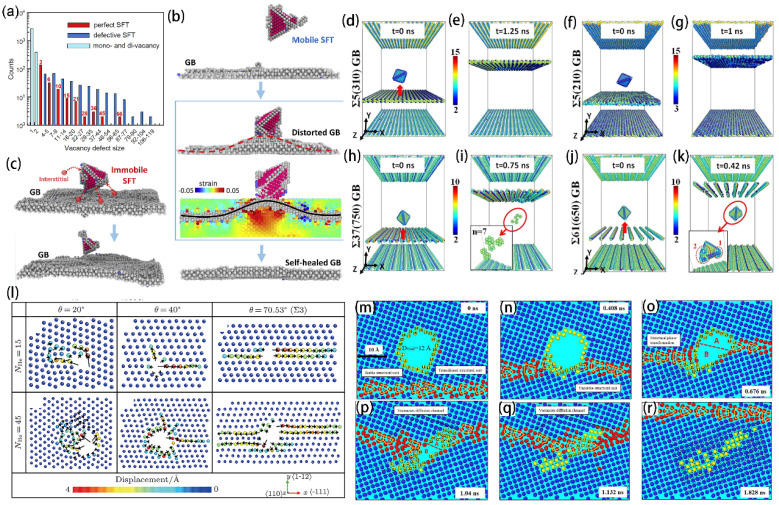
Cu serves as a benchmark FCC system for elucidating SFT–GB interactions, whereas W models He-bubble dynamics at GBs in fusion-relevant refractory environments. (**a**) Statistical distribution of vacancy-type defect sizes in irradiated ∑5(210) Cu with grain spacing of 11.4 nm at damage dose from 0.06 dpa to 0.27 dpa. (**b**) SFT annihilation process via interaction with a GB. (**c**) SFT annihilation by interstitial recombination [185]. Snapshots of the atomic images before and after the interaction between SFT and GBs at 10 K, (**d**–**g**) Σ37(750) GB, and (**h**–**k**) ∑61(650) GB [186]. (**l**) Displacements of W atoms in the GB plane where He bubbles are located. Arrows represent direction of displacement. Atoms are colored according to magnitude of displacement as indicated by color bar. View is along the [110] direction [189]. (**m**–**r**) Bottom view of the interaction process between ∑5 (310) GB and D12 voids. The disordered regions on GB after its interaction with voids are indicated by the yellow dotted line [191]. Reproduced with permission from Refs. [185,186,189,191].

The absorption of defects by GBs is not limited to interstitial atoms, but also exerts a certain absorption effect on SFT, bubbles, and voids. As is well known, most SFT defects are immobile and stable, and their energy stability increases with size [182,183]. However, there are also some SFTs that are quite mobile, and their diffusivities can be even higher than that of mono-vacancies [184]. Therefore, investigating the mechanism underlying the interaction between SFTs and GBs enables a deeper understanding of the irradiation damage resistance strategies for alloys. Importantly, Jin et al. [185] found that irradiation-induced SFTs fall into two categories, with over 82% being high-mobility defective SFTs and less than 18% being low-mobility perfect SFTs (Figure 12a) and systematically identified two typical annihilation modes of SFTs in nanocrystalline Cu: (1) Removal by GBs (Figure 12b). The SFT also prefers to migrate toward the GBs filled with interstitial atoms, and then the GB atoms rearrange to accommodate the SFT. The distorted GBs are in direct contact with SFT, indicating an attractive interaction between the irradiated GBs and SFT [186]. After the SFT annihilation, the GBs returned to a straight shape, demonstrating an elastic self-healing response to radiation damage [187]. (2) Removal by GBs (Figure 12c). In this process, there is no direct interaction between SFTs and GBs. But the size of SFTs still decreases and eventually vanishes, primarily due to the absorption of interstitial atoms in SFTs by GBs. The process of the direct absorption of interstitial atoms in SFTs has also been reported in nanocrystalline Ni [188]. It is worth noting that the occurrence of these two modes depends on the mobility of the corresponding SFTs. Building on this, Zhang et al. [186] demonstrated in Figure 12d–k that shear-coupled GB migration actively sweeps and absorbs stationary SFTs with an efficiency dictated by GB structure. High-angle grain boundaries (HAGBs) facilitate total SFT annihilation even at extremely low temperatures while low-angle grain boundaries (LAGBs) exhibit markedly reduced efficiency. This work identifies a new interaction pathway between GBs and SFTs and complements previous studies focused on static GBs capturing mobile defects.

In addition, Li et al. [189] investigated the nucleation and growth of He bubbles at GBs in W and observed that the displacement along the direction of dislocation junctions induces changes in the local GB structure, leading to the formation of partially ordered GBs in the [−111] dislocation bands (Figure 12l). Qin et al. [190] used a thermodynamic model to analyze the nucleation of intergranular He bubbles, and the results showed that the nucleation of He bubbles is closely related to the GB structure, and He bubbles are mainly formed on HAGBs. This is attributed to the fact that HAGBs have much higher excess grain boundary energy and atomic free volume than LAGBs, enabling them to serve as preferential sites for the heterogeneous nucleation of He bubbles and significantly reduce the critical nucleation work required for the formation of He–vacancy clusters. It is worth noting that there remains a lack of a quantitative relationship between GB energy and the critical nucleation threshold of He bubbles at present. Critically, this lack of energetic correlations highlights a fundamental limitation where short nanosecond simulation windows fail to capture the structural transitions and stress fields evolving under realistic long-term reactor conditions. To overcome this temporal bottleneck, Liu et al. applied parallel replica AMD to achieve extended timescales in tungsten grain boundaries. Their long-duration modeling revealed an unprecedented pressure-driven asymmetric bubble growth mode where stress induced grain boundary shearing operates as a structural latch rather than conventional isotropic expansion. GBs also exert a certain absorptive effect on voids, during which the voids exert resistance on migrating GBs and alter their local structure. Zhang et al. [191] studied the void dissolution mechanism at the migrating Σ5 (310) GB at 400 K. Their study revealed that once the GB comes into contact with the void, atoms at the void surface adjacent to the GB undergo atomic rearrangement, resulting in a corresponding reduction in void size, and that a structural phase transformation occurs at the GB simultaneously, where the stable structural unit a transforms into a new atomic configuration b with higher energy (Figure 12m–r).

In summary, CSRO and GBs are currently the two primary strategies to enhance the irradiation resistance of alloys, and the mechanisms by which they alleviate irradiation damage differ. On the one hand, CSRO primarily suppresses the generation and migration of point defects during irradiation by modulating the energy barriers for defect migration and formation, thereby enhancing the irradiation resistance of the alloy. The defect annihilation efficiency of GBs is intrinsically determined by their crystallographic characteristics (e.g., misorientation angle and Σ value) that govern the energy barrier of GB migration and atomic rearrangement, and is further extrinsically modulated by temperature, applied stress, and defect mobility that regulate the kinetics of GB-defect interactions.

## 5. Effect of Radiation on Mechanical Properties

The mechanical consequences of irradiation are governed not only by irradiation dose, but also by the type, size, number density, spatial distribution, and stability of the defect population generated during the primary-damage and defect-evolution stages described in Section 2 and Section 3. The defect-specific mechanical mechanisms are summarized in Table 4. Isolated point defects can modify local lattice strain, diffusion, and dislocation nucleation, whereas their aggregation produces clusters, dislocation loops, SFT, voids, and gas bubbles that act either as obstacles to dislocation glide or as cavities that promote damage localization. Consequently, irradiation may cause hardening and loss of ductility, while local softening or premature yielding can also occur when defects facilitate dislocation nucleation or cavity growth.

**Figure 13:**
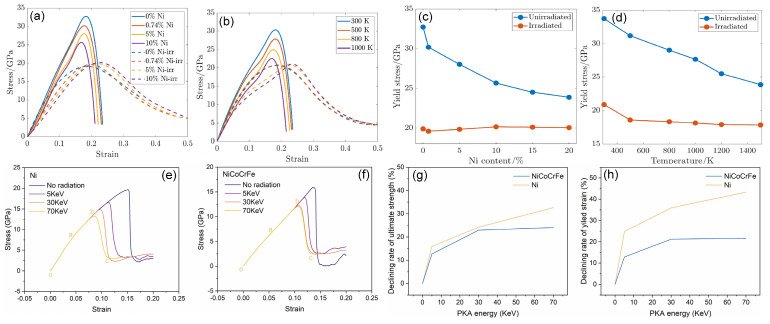
Fe-Ni is used as a dilute Fe-based structural-alloy model, whereas NiCoCrFe is a compositionally complex alloy model. (**a**) The unirradiated and irradiated stress–strain curve under different Ni content. (**b**) The unirradiated and irradiated stress–strain curve under different temperatures for Fe-0.74 wt.% Ni alloy. (**c**) The unirradiated and irradiated yield stress curve under different Ni content. (**d**) The unirradiated and irradiated yield stress curve under different temperatures for Fe-0.74 wt.% Ni alloy [199]. (**e**,**f**) Stress–strain curves of tensile tests after different energy irradiation and (**g**,**h**) the declining rate of ultimate strength and yield strain [200]. Reproduced with permission from Refs. [185,186].

The presence of high-throughput and high-energy incident particles is considered a dominating factor for the degradation of material performance [173,192,193]. During irradiation, the formation of defects such as FPs, dislocation loops, and SFTs severely impairs the mechanical properties of nuclear structural materials, leading to phenomena including hardening [194,195] and embrittlement [196,197], among others. The performance of alloys during irradiation is influenced by their chemical compositions, primarily due to the fact that alloy compositions affect the generation of freely migrating point defects as mentioned earlier. Zheng et al. [10] studied the effects of alloy composition on the tensile properties of Fe-Cr-Al alloys before and after irradiation. The elastic modulus of both unirradiated and irradiated alloys decreases with the decrease in Cr content, and then increases above Cr 15.13% (Fe-15Cr-5Al), indicating that the tensile properties of the alloys after irradiation are closely related to the Cr content. Dai et al. [198] reported that when the Cr content is constant, the addition of Al enhances the elastic properties of Fe-Cr-Al alloys by strengthening atomic bonding. He et al. [199] conducted MD simulations of Fe-Ni alloy based on MLP and investigated the synergistic effect of solute element content and temperature on its tensile behavior during irradiation. Compared with unirradiated conditions, the tensile stress and yield strain of Fe-Ni alloys decreased after irradiation, due to Ni diffusion via a vacancy mechanism accelerating void formation, easing plastic deformation, and reducing required stress (in Figure 13a–d). The tensile stress change in Fe-Ni alloys after irradiation is not significant, which is attributed to the uniform distribution of Ni atoms in the Fe-Ni alloy, and this diffusion provides a cushioning effect during the tensile deformation process. Yu et al. [200] used a higher irradiation energy (70 keV) than in previous HEA irradiation simulation studies to investigate the changes in tensile properties of NiCoCrFe HEA and compared it with Ni. They discovered that the stress–strain curves have no significant difference before yield, but the yield point and yield strength obviously decrease with the increase in irradiation energy (in Figure 13e–h). As tensile strain is applied, the point defects induced by cascades in the alloy become nucleation sites for dislocations [201,202]; this ultimately leads to premature plastic deformation and a reduction in yield strength, with the effect becoming more pronounced as the cascade energy increases [41].

**Figure 14:**
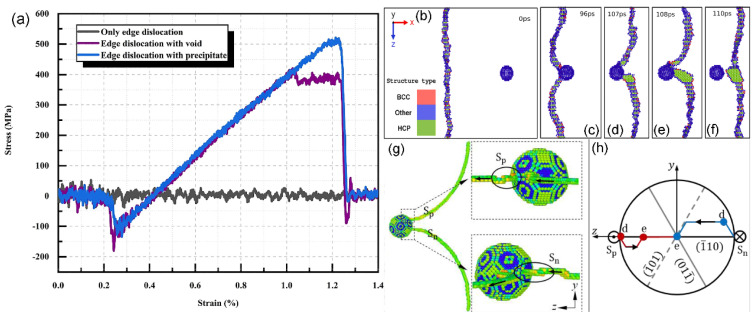
Ni models precipitate and void obstacles in FCC systems; Fe–Ni–Cr illustrates void dynamics in high-temperature structural alloys, and W demonstrates void-induced hardening in refractory metals. (**a**) Stress–strain curves of the interactions of edge dislocations without and with defects [208]. (**b**–**f**) Snapshots of the interaction between an edge dislocation and a void (with a diameter of 2 nm) are presented, illustrating the occurrence of the Friedel effect [213]. (**g**,**h**) Typical atomic structures of dislocation–void configurations during shear deformation and illustration of the trajectory of negative and positive screw arms along the void surface viewed along the *x*-axis. Black arrows on the line indicate the move direction of the arms [214]. Reproduced with permission from Refs. [208,213,214].

The extended defects described in Section 3 exert a more direct influence on plastic deformation. Dislocation loops produce long-range stress fields and impede dislocation glide, thereby increasing the critical resolved shear stress and contributing to irradiation hardening; their strengthening effect depends on loop type, size, number density, and solute decoration. In FCC materials, stacking-fault tetrahedra are sessile vacancy-type defects that also obstruct mobile dislocations and may promote localized slip under high stresses. Therefore, dislocation loops and SFTs should be considered as defect-specific strengthening and embrittlement contributors rather than being included only in the generic term “irradiation defects”. Irradiation hardening arises when stable defect clusters, dislocation loops, SFT, radiation-induced precipitates, voids, and fine He bubbles act as obstacles to dislocation motion. It has been reported that irradiation hardening in Fe-Cr-Al alloys is primarily caused by Cr-rich precipitation [199], and high Cr content promotes the precipitation of Cr rich α phases [203,204], which dominates radiation hardening [205]. Krasnikov et al. [206] reported via the DDD method that second phases in Al-Cu alloys enhance the yield strength, and this process is correlated with the size of the second phases. Furthermore, Liao et al.’s study mentioned that the effect of this Cr-rich precipitate on radiation hardening is closely related to its diameter [207], and 2 nm precipitates can be completely dissolved under 30 keV, while larger precipitates (3 and 5 nm in diameter) exhibit only minor changes and thus affect the mechanical properties. Cui et al. [208] employed MD simulations to investigate the interactions between edge dislocations and irradiation-induced defects (void and Ni_3_Al precipitates) in nickel. With both the void and Ni_3_Al precipitates set to a size of 3 nm, a gradually increasing shear stress was applied to overcome lattice resistance, revealing that the critical resolved shear stress (CRSS) of both defects is significantly higher than that of the defect-free Ni matrix (Figure 14a). Such an increase in CRSS also serves as one of the contributing factors to the irradiation hardening of the alloy. Furthermore, the CRSS during the interaction process increases with the increase in defect size [209,210,211], while decreasing with the rise in temperature [212].

Investigating the interaction mechanisms between voids and dislocations facilitates an understanding of irradiation hardening mechanisms. Radiation-induced voids are located within the crystal, and there are two possibilities for dislocations to encounter the void: the shear mechanism (known as the Friedel effect) or forming dislocation loops through the Orowan mechanism. Previously, Lee et al. [215] reported that regardless of the void size, the interaction between screw dislocations and voids with a diameter of 1.5–5 nm follows the shear mechanism. Chen et al. [216] further advanced the research on voids under the shear mechanism; they considered arbitrary void geometries under various irradiation doses and temperatures and predicted the CRSS of dislocations interacting with arbitrary void geometries. Figure 14b–f shows the Friedel effect in FeNiCr alloys [213]. Under the action of applied stress, the dislocation line bows when it encounters a void. The dislocation segment fixed on the cavity will resist further slip, and as the deformation continues to increase, the dislocation bows until it breaks away at the CRSS. Yu et al. [214] reported that the interaction process between dislocations and voids generates a helical dislocation dipole with a length of 3b (Figure 14g), where b is the magnitude of the Burgers vector. When the stress increases to its maximum value, the two screw arms can recover to their original (110) plane through double cross-slip (Figure 14h). For the Orowan mechanism, Bacon et al. [217] proposed the Bacon–Kocks–Scattergood (BKS) model early on, which takes into account the effects of dislocation self-interactions and the random distribution of irradiation obstacles. Subsequently, Vaid et al. [218] further modified the BKS equation by incorporating solute hardening in solid solutions, enabling quantitative prediction of the CRSS in solid solutions with voids and its dependence on void spacing.

**Figure 15:**
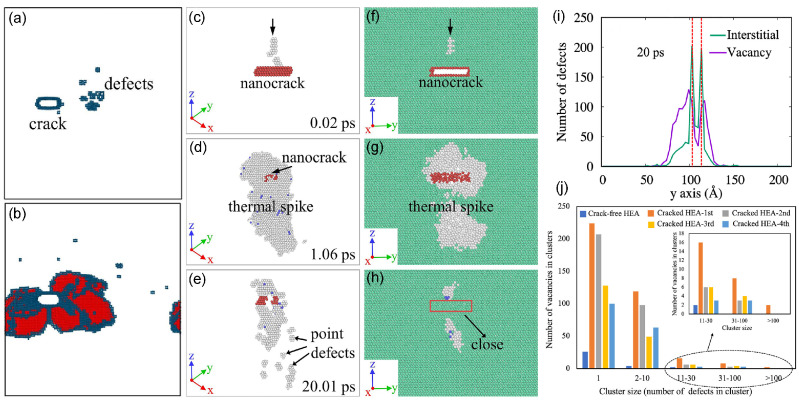
Ni, α-Zr, and FeCoCrNiAl model distinct irradiation responses, crack-tip deformation (FCC), cladding dynamics (HCP), and crack healing (complex alloys), respectively. The deformation characteristics of the crack tip for irradiated Ni at 300 K from cycle 9 to cycle 10: (**a**) 114 ps and (**b**) 127 ps [220]. The evolution of the collision cascades at different times upon 10 keV E_PKA_. The distance between the crack surface and the PKA is 32 Å, and the PKA direction is perpendicular to the crack surface. (**c**–**e**) The 3D snapshots with hcp atoms erased for better visualization of the irradiation defects, and (**f**–**h**) the corresponding cross-section snapshots [221]. (**i**) Spatial distribution of defects in the cracked HEA at 20 ps at the first collision cascade. (**j**) Number of clusters with different sizes in the crack-free HEA at the first cascade in comparison to the cracked HEA at four overlapping cascades [222]. Reproduced with permission from Refs. [220,221,222].

Crack propagation is concealed, yet at elevated temperatures, cracks provide rapid diffusion channels for atoms to accelerate alloy creep failure and degrade mechanical properties. Previous studies have reported that irradiation has been demonstrated to suppress the propagation of nanoscale cracks within the alloy matrix, which consequently contributes to the enhancement of the fracture toughness of the alloy. Fuller et al. [219] estimated the fatigue crack length based on the fatigue crack propagation model of a strip-yield and extended it to the irradiation effect. Ma et al. [220] used MD simulations to insert a crack into Ni single crystals before irradiation and study the behavior of crack propagation under cyclic loading for irradiated Ni. At 300 K, cracks begin to appear and exhibit blunting at the atomic scale, and irradiation defects gradually move toward the crack tip (Figure 15a,b). Given this, Wang et al. [221] conducted MD simulations to investigate the interaction between collision cascades and nanoscale cracks in Zr (Figure 15c–h). They found that when thermal spikes overlap with nanoscale cracks, collision cascades induce the healing of nanoscale cracks, as shown in Figure 15h; this effect is recognized to effectively enhance the fracture toughness of the alloy. Recently, Xu et al. [222] selected the FeCoCrNiAl alloy to investigate the irradiation behavior of pre-cracked high-temperature alloys and revealed the crack healing mechanism by analyzing microstructural evolution and defect interactions. Observations of defect counts on cracks during thermal equilibrium reveal that numerous atoms migrate into cracks or recombine, reducing the number of interstitial atoms in the matrix (in Figure 15i). Furthermore, compared with the first cascade, the number of medium-sized vacancy clusters was much lower, and no large vacancy clusters were observed in the subsequent three cascades (see Figure 15j).

The mechanical response to irradiation is not universal but is determined by the defect population and its interaction with dislocations and cracks. Defect clusters, dislocation loops, SFT, fine He bubbles, and radiation-induced precipitates commonly increase flow stress by impeding dislocation glide, while void growth and large or intergranular He bubbles promote swelling, damage localization, loss of ductility, and fracture. At the atomic scale, the overall mechanical response results from the competition between dislocation–obstacle interactions and defect-assisted cavity growth or crack-tip atomic rearrangement. Translating atomistic interactions into engineering-scale predictions requires rigorous multiscale homogenization. MD extracts fundamental obstacle strengths and critical resolved shear stresses (CRSSs) for specific defects. DDD then utilizes these parameters to simulate collective dislocation dynamics and strain localization. Finally, crystal plasticity and FEM predict component-level responses via irradiation-dependent hardening laws. However, continuum accuracy hinges on robust constitutive calibration and the proper representation of defect heterogeneity. Thus, atomistic mechanisms must undergo mesoscale upscaling and experimental validation before macroscopic application.

As mentioned above, atomic simulation studies on the irradiation damage behavior of alloys have mainly focused on tensile and fracture toughness properties. The current mainstream conclusion is that irradiation will reduce the yield strength of alloys and advance the plastic point, thereby degrading their mechanical properties. At the atomic scale, irradiation hardening originates primarily from the elevated CRSS induced by dislocation–defect interactions following either the shear mechanism (Friedel effect) or the Orowan mechanism. The irradiation-enhanced fracture toughness of the alloy is attributed to collision cascade-driven atomic rearrangement within the alloy matrix, thereby filling the pre-existing nanoscale cracks. These mechanistic insights provide critical theoretical guidance for the rational design of high-performance irradiation-tolerant structural alloys.

## 6. Summary and Outlook

Computational simulations provide theoretical interpretation, complement experimental observations, and accelerate the design of radiation-resistant metallic materials. They also reveal irradiation-damage mechanisms and help link atomistic defect processes to the macroscopic mechanical response of nuclear structural materials. This review first summarizes the defect generation in metallic materials during irradiation and shows that defect clusters formed through the migration and aggregation of Frenkel pairs can accumulate and intensify irradiation damage. The differences in the size, number density, and type of defects are governed by the PKA direction, *E_PKA_*, temperature, and strain, as well as crystal structure, alloy chemistry, and microstructural sinks. Subsequently, we discuss the evolution and self-healing mechanisms of irradiation-induced defects. The nucleation, growth, and stability of dislocation loops, voids, and He bubbles are controlled by defect-migration and recombination barriers, dislocation dynamics, and sink effects. The stability of He bubbles is also strongly modulated by the Vac/He atomic ratio in the alloy. As summarized in Table 3 and Table 4, the defect-evolution pathways are material-dependent; BCC Fe- and W-based materials, FCC Ni- and Fe–Ni–Cr-based alloys, HCP Zr alloys, and compositionally complex alloys exhibit distinct characteristic defect populations and kinetics. The CSRO can modify defect-formation and migration energies and thereby alter defect trapping, recombination, and clustering, while GBs can act as effective defect sinks depending on their crystallographic character and local atomic structure; both processes involve atomic rearrangement within defect structures. Finally, the review generalizes the dual effects of irradiation on the mechanical properties of alloys and their underlying atomic-scale origins, including irradiation hardening induced by dislocation–defect interactions and irradiation-enhanced fracture toughness driven by atomic rearrangement.

Although computational simulation studies have yielded abundant results in understanding the formation and evolution mechanisms of irradiation-induced structural defects in nuclear structural materials, several challenges remain. Firstly, standardized simulation protocols are required for more realistic irradiation scenarios. For example, developing a robust theoretical framework for energy dissipation during external PKA incidence would reduce systematic uncertainties in atomistic simulations. In addition, exploring the anisotropic evolution laws of TDE under coupled thermal, mechanical, and irradiation fields, and constructing material-specific databases for various alloy categories, remain important. Second, highly accurate and transferable atomic potentials for irradiation simulation should be developed. Based on datasets generated from high-throughput DFT calculations, machine learning potential frameworks can be used to train atomic potentials with DFT-level accuracy and high computational efficiency. Well-trained potentials can provide a high-fidelity physical basis for atomic-scale simulations of displacement cascades and improve predictions of irradiation damage. Third, multiscale integration is required for describing long-term defect kinetics. Current simulation studies on irradiation damage are mostly focused on MD simulations and first-principle calculations, which can only cover picosecond or nanosecond timescales and merely simulate the generation of initial defects from irradiation-induced displacement cascades. Therefore, it is urgent to introduce higher-scale simulation methods, such as phase-field simulation and the finite element method, to describe long-range diffusion, aggregation, and recombination, as well as the long-term evolution behavior of irradiation-induced defects. Finally, it is also urgent to carry out mutual verification between computational simulation and structural characterization experiments. With advanced characterization techniques including in situ transmission electron microscopy and synchrotron radiation-based characterization methods, it is possible to simultaneously observe the atomic-scale defect evolution and macroscopic performance degradation during irradiation, and establish a quantitative mapping model between atomic-scale defect evolution and the macroscopic service performance of materials.

## Figures and Tables

**Table 1:** Comparison of representative computational methods, accessible time/length scales, advantages, limitations, and computational costs for irradiation-induced defect simulations in metallic materials.

Method	Time/Scale	Software	Main Output	Advantages	Limitation	Cost
DFT	ps–ns Å–nm	VASP, Materials Studio	defect formation/migration energy	high accuracy/electronic effects	limited system size	very high
MD/TAD	ps–ns nm–µm	LAMMPS	cascades/Frenkel pairs/clusters	cascade dynamics/large-scale	short timescale	high
SRIM/TRIM	nm–mm	SRIM	implantation/recoil profile	transport/damage profiles	lacks atomistics	low
OKMC	ns nm–µm	MMonCa	diffusion/clustering kinetics	macro-times/spatial defects	parameter-dependent	moderate
CD/Rate theory	year	custom codes	defect population evolution	long-term/high dose	ignores local heterogeneity	low
DDD	ns–ms µm–mm	ParaDiS/custom codes	dislocation/microstructure	mechanical behavior	Lower scale/calibration	moderate to high
Phase field	year µm–mm	Moose	void/precipitate	interface evolution	lower-scale inputs	moderate to high
MLP-MD	fs–ns nm–µm	DeePMD/GPUMD	cascades/Frenkel pairs/clusters	DFT-level accuracy	data dependency	high to very high

**Table 2:** Representative interatomic potentials and reported TDEs in metallic materials. (TDE values are quoted for the representative potential–material combinations in the cited studies rather than for the potential framework itself; “dir.”, “min.”, and “avg.” denote the directional range, minimum value among sampled recoil directions, and orientation-averaged value, respectively).

Potential Framework	Representative Systems/Models	Reported TDE (eV)	Main Advantages	Main Limitations	References
EAM/FS	α-Fe,Fe-based alloys and W	Fe:20–100 (dir); W: 41–98	Fast; widely used for TDE and cascades	Potential-sensitive; Short-range corrections required	[66,67,68,69,70]
MEAM/ADP	Al, Pu, U alloys, CoCrFeMnNi	Al: 22.67 (avg.); Al_3_U: 20–41; HEA: 16–64 (dir)	Suitable for alloys and competing phases	Uncertain cascade transferability	[23,71,72]
DP–ZBL	Al	26.54 (avg.; recommended: 25)	DFT-level fitting with short-range repulsion	Sensitive to training data	[39]
GAP/tabGAP	W, Ta, W–Mo	Mo/Ta/W: 31/35/43 (min.); HEA: 44.3–48.6 (avg.)	Accurate defect description; relatively efficient	Higher cost than EAM; limited by training domain	[73,74]
NEP–ZBL/UNEP	W, MoNbTaVW, MoTaVW	40–50 (avg., model dependent)	Efficient large-scale and multicomponent simulations	Requires extensive short-range training data	[61,75,76]

**Table 3:** Representative metallic materials considered in irradiation-damage simulations, their commonly observed irradiation-induced defects, and relevance to this review.

Material	Typical Application	Crystal Structure	Typical Irradiation Defects	Relevance
Fe/Fe–Cr/Fe–Cr–Al	fission structural/cladding candidates	BCC	<111>/<100> loops, vacancy clusters/voids and α′ precipitation	major nuclear structural alloys
Austenitic/Ni-based alloys	high-temperature reactor components	FCC	faulted/perfect loops, SFTs, cavities, segregation	classical high-temperature alloys
W/W alloys	plasma-facing fusion components	BCC	interstitial loops, bubbles	fusion relevance
Zr alloys	fuel cladding	HCP	basal/prismatic loops, irradiation growth	LWR relevance
HEAs/CCAs	candidate materials	FCC/BCC/multiphase	defect clusters, loops, and cavities	chemical-disorder effects
Cu/Ni/Fe	model systems	FCC/BCC	vacancy/interstitial clusters, loops, and SFTs	mechanism benchmarking

**Table 4:** Defect-specific effects of irradiation-induced structural defects on the mechanical properties of metallic materials.

Defect Type	Principal Mechanical Consequence	Atomistic Mechanism	Representative Systems
Vacancies, SIAs, and Frenkel pairs	Local softening or premature yielding	Local lattice distortion and dislocation nucleation;	α-Zr, Fe–Ni, Fe-based alloys
Vacancy/interstitial clusters	Early-stage hardening	Local stress fields; embryos for loops, SFTs, and cavities	FeCrAl, W-based alloys, CCAs
Dislocation loops	Irradiation hardening, reduced ductility, crack-initiation sites	Obstacles to dislocation glide; increased CRSS	FeCrAl, Fe–Cr, W, Ni-based alloys
SFTs	Hardening and possible slip localization	Vacancy-type obstacles interacting with dislocations	Cu, Ni, Fe–Ni–Cr alloys
Voids	Small voids: hardening; large/dense voids: swelling, creep, ductility loss, and fracture	Friedel/Orowan interactions; cavity growth and coalescence	W, Fe–Ni–Cr, HEAs
He bubbles	Overpressurized or intergranular bubbles: swelling and embrittlement	Dislocation pinning and internal pressure	W, Fe-based steels, Ni/Cu model systems
Cr-rich α′ precipitates	Hardening, especially in FeCrAl	Dislocation pinning by solute-rich nanoscale obstacles	FeCrAl alloys

## Data Availability

No new data were created or analyzed in this study. Data sharing does not apply to this article.
